# Supplementary material for: Synthesis of novel N-substituted β-amino acid derivatives bearing 2-hydroxyphenyl moieties as promising antimicrobial candidates targeting multidrug-resistant Gram-positive pathogens
Source: PLoS One. 2025 Jun 12;20(6):e0311715. doi: 10.1371/journal.pone.0311715 (PMC12161524; doi:10.1371/journal.pone.0311715)
Supplement: S1 File — 1H and 13C NMR spectra of compounds 2 − 26 (in DMSO-d6). Table S1. The in vitro antimicrobial activity of N-substituted β-amino acid derivatives 2–26 against panel of fungal pathogens. (DOCX) [file pone.0311715.s001.docx]

**Synthesis of** **Novel** ***N*-Substituted *β*-Amino Acid Derivatives Bearing 2-Hydroxyphenyl Moieties as Promising Antimicrobial Candidates Targeting Multidrug-Resistant Gram-Positive Pathogens**

**Povilas Kavaliauskas^1,2,3,4*^; Birutė Grybaitė^1^; Birute Sapijanskaite-Banevič^1^; Rūta Petraitienė^2,3^; Ramunė Grigalevičiūtė^4,5^; Andrew Garcia^2^; Ethan Naing^2^; Vytautas Mickevičius^1^&, Sergey Belyakov ^7^, and Vidmantas Petraitis^2,3,6^&.**

**Supporting information**

**Figure S1-S50.** ^1^H and ^13^C NMR, ^19^F NMR spectra of compounds **2−26** (in DMSO-*d_6_*)

**Table S1.** The *in vitro* antimicrobial activity of *N*-substituted *β*-amino acid derivatives **2-26** against panel of fungal pathogens.

**Table S2.** Crystal data and structure refinement for compound **24** (named BGiii159).

**Table S3**. Fractional Atomic Coordinates (×10^4^) and Equivalent Isotropic Displacement Parameters (Å2×103) for compound **24** (named BGiii159). Ueq is defined as 1/3 of of the trace of the orthogonalised UIJ tensor.

**Table S4.** Anisotropic Displacement Parameters (Å2×103) for compound **24** (named BGiii159). The Anisotropic displacement factor exponent takes the form: -2π2[h2a*2U11+2hka*b*U12+…].

**Table S5.** Bond Lengths for compound **24** (Named BGiii159).

**Table S6.** Bond Angles for compound **24** (Named BGiii159).

**Table S7.** Torsion Angles for compound **24** (Named BGiii159)**.**

**Table S8.** Hydrogen Atom Coordinates (Å×104) and Isotropic Displacement Parameters (Å2×10^3^) for compound **24** (Named BGiii159).

*3,3'-((2-Hydroxyphenyl)azanediyl)dipropionic acid* ***(2)***

**Figure S1**. ^1^H NMR spectrum of compound **2**


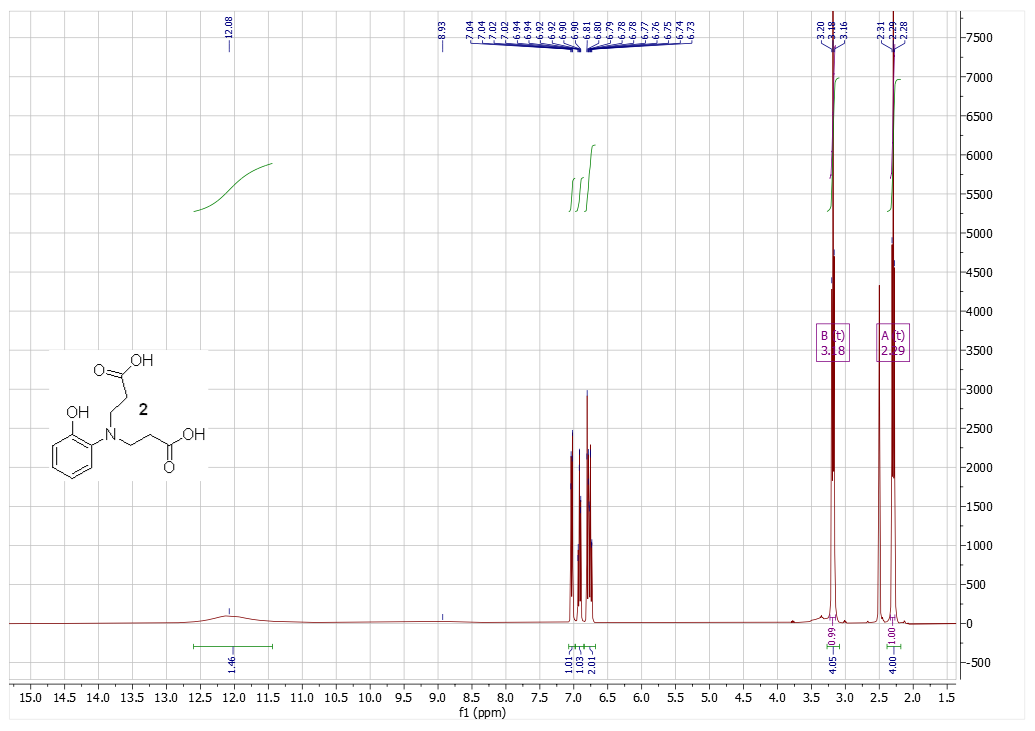


**Figure S2**. ^13^C NMR spectrum of compound **2**


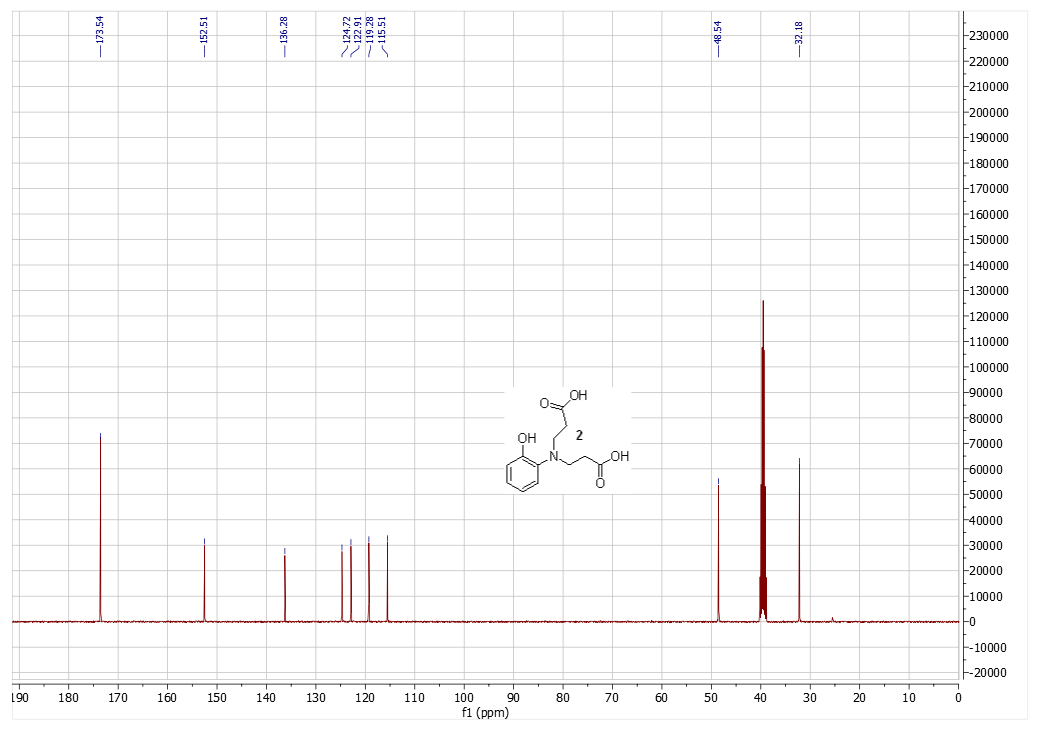


*3-(2-oxo-3,4-dihydrobenzo[b][1,4]oxazepin-5(2H)-yl)-N-(4-sulfamoylphenyl)propenamide* ***(3)***


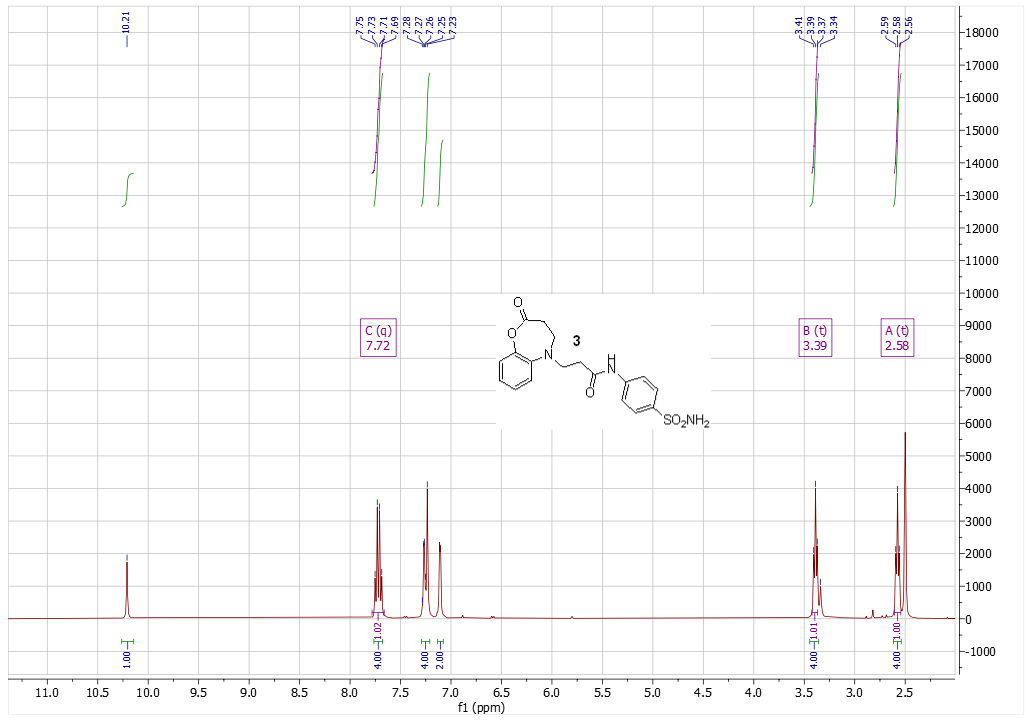


**Figure S3**. ^1^H NMR spectrum of compound **3**

**
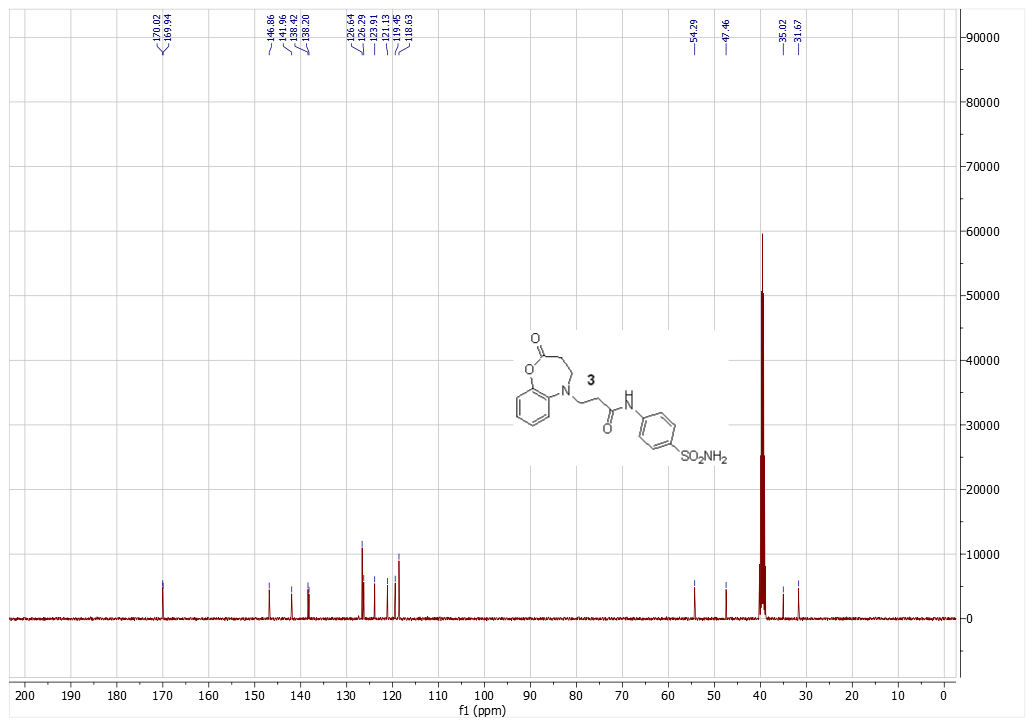
**

**Figure S4**. ^13^C NMR spectrum of compound **3**

*Dimethyl 3,3'-((2-hydroxyphenyl)azanediyl)dipropionate* ***(4)***

***
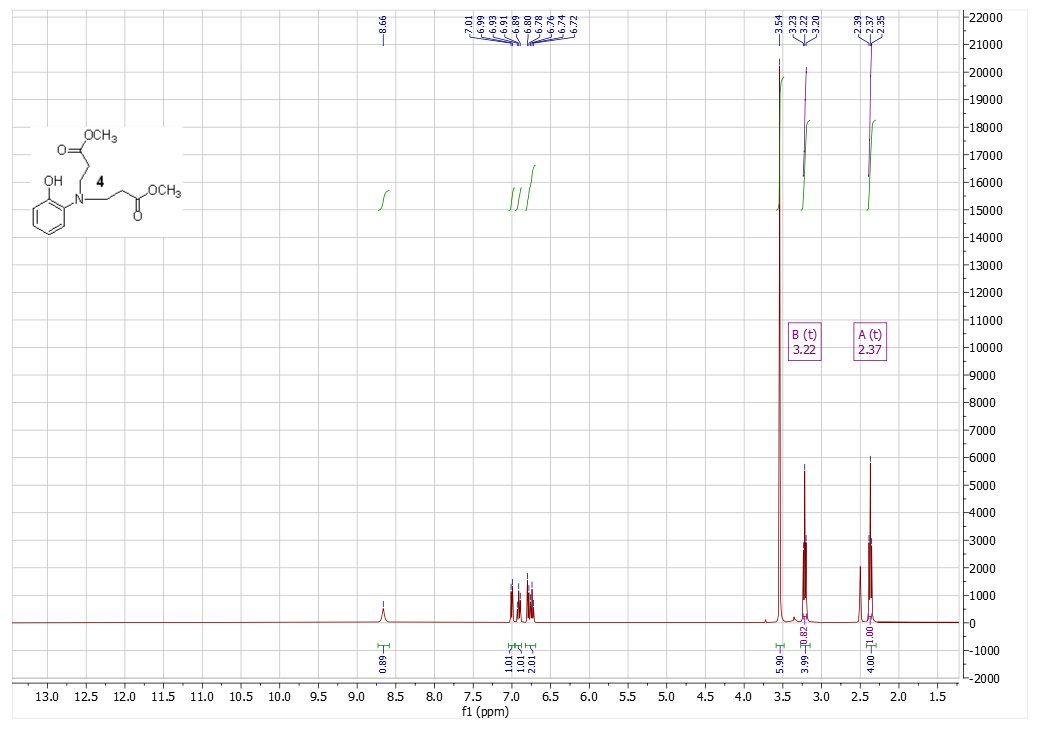
***

**Figure S5**. ^1^H NMR spectrum of compound **4**

**
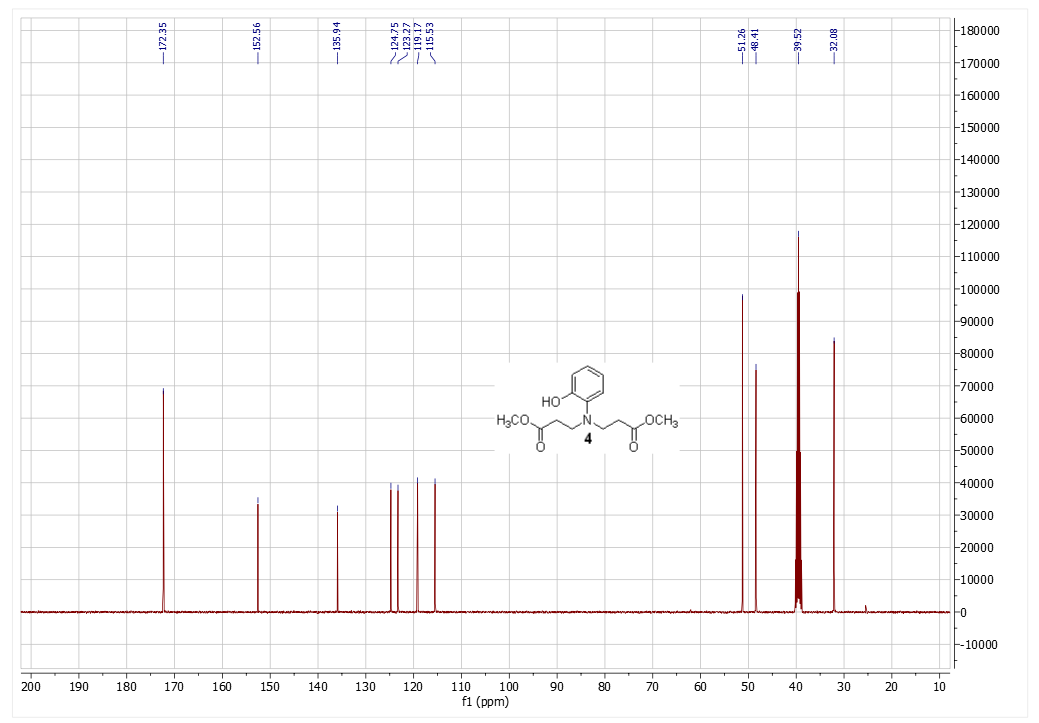
**

**Figure S6**. ^13^C NMR spectrum of compound **4**

*3,3'-((2-Hydroxyphenyl)azanediyl)di(propanehydrazide)* ***(5)***

*
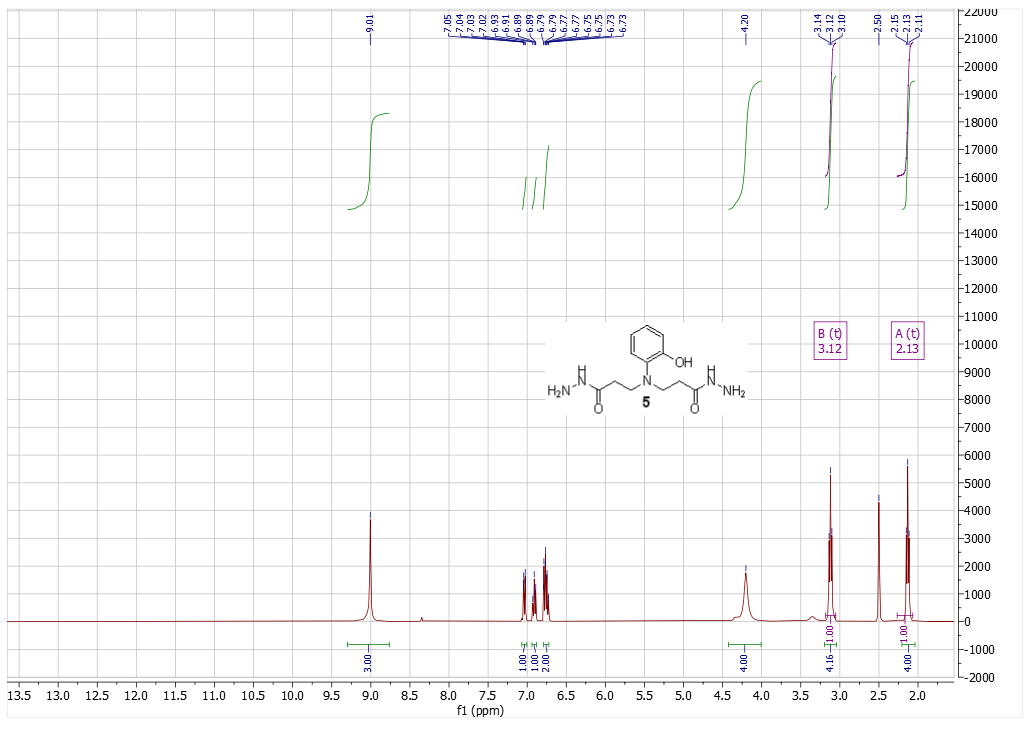
*

**Figure S7**. ^1^H NMR spectrum of compound **5**

**
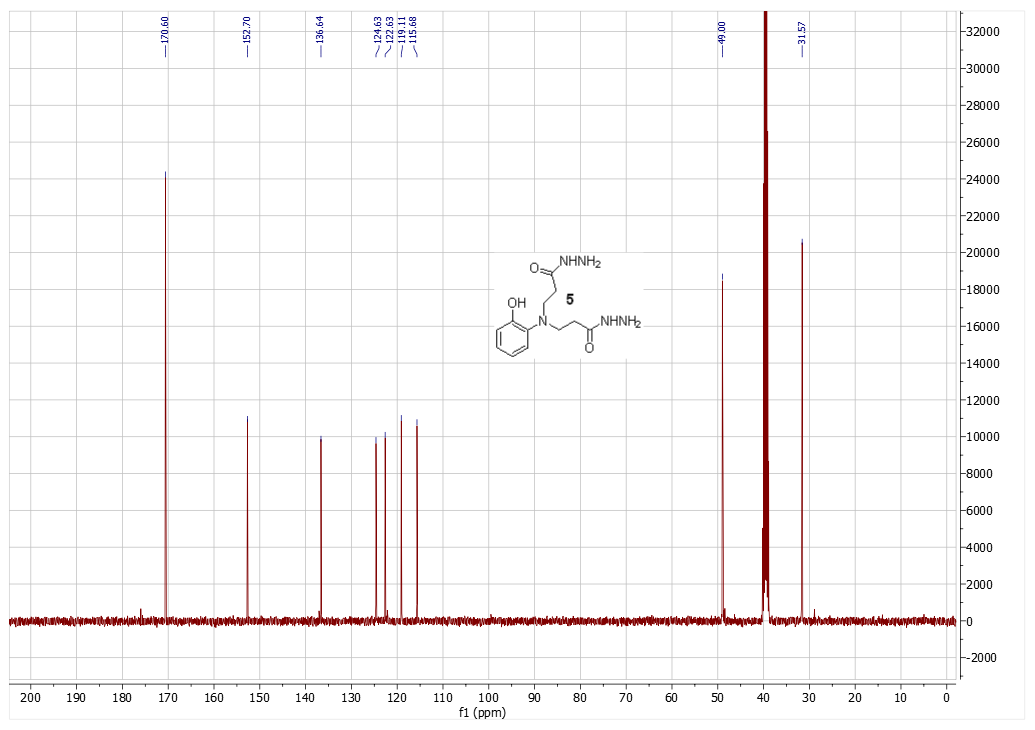
**

**Figure S8**. ^13^C NMR spectrum of compound **5**

*2-((2-(1H-benzo[d]imidazol-2-yl)ethyl)amino)phenol* ***(6)***

*
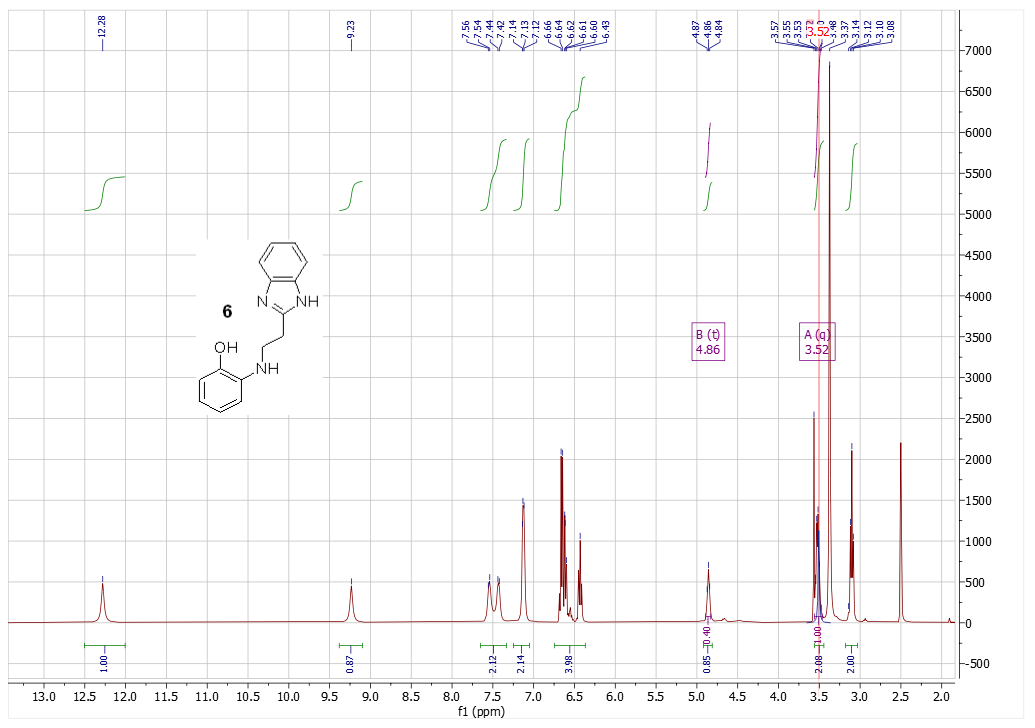
*

**Figure S9**. ^1^H NMR spectrum of compound **6**

**
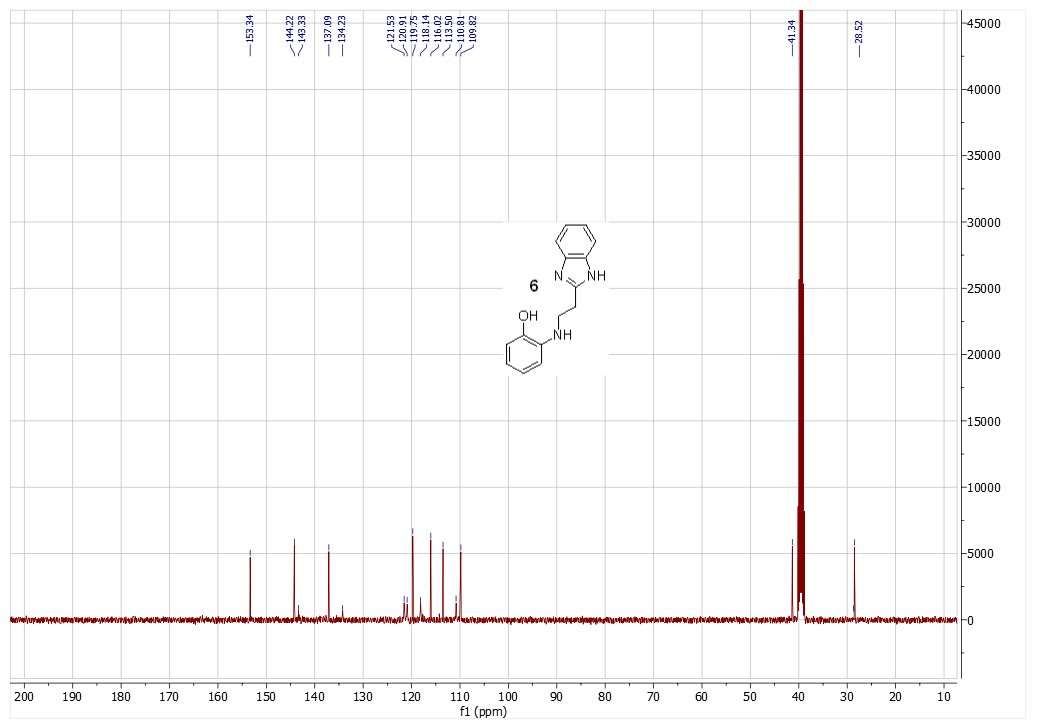
**

**Figure S10**. ^13^C NMR spectrum of compound **6**

*3,3'-((2-hydroxyphenyl)azanediyl)bis(N'-(benzylidene)propanehydrazide)* ***(7)***

***
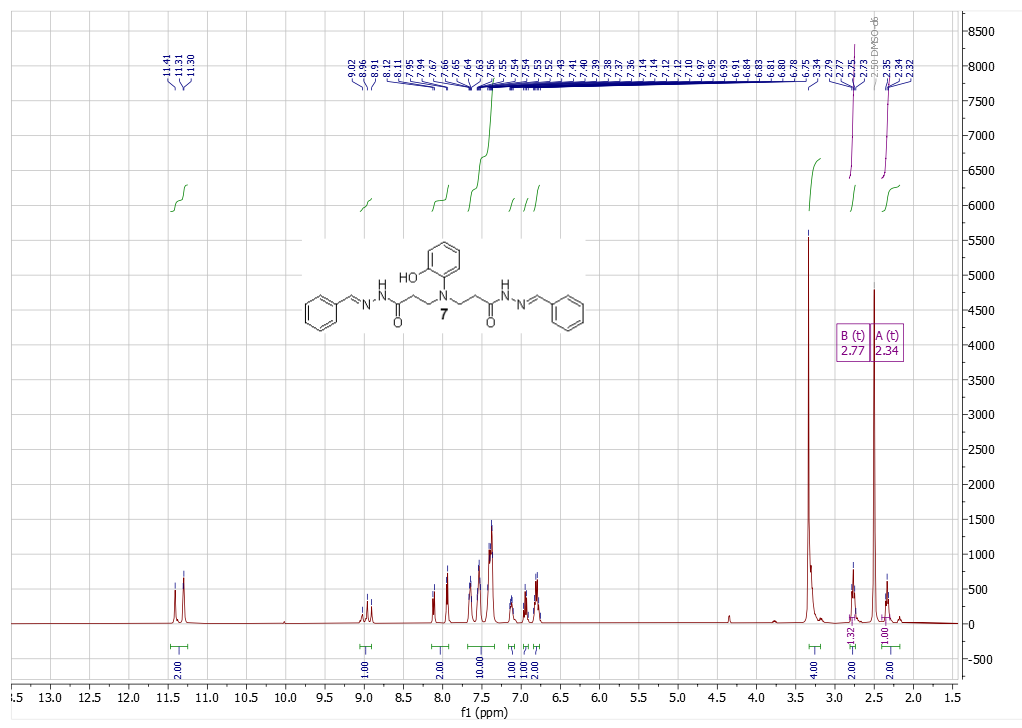
***

**Figure S11**. ^1^H NMR spectrum of compound **7**

**
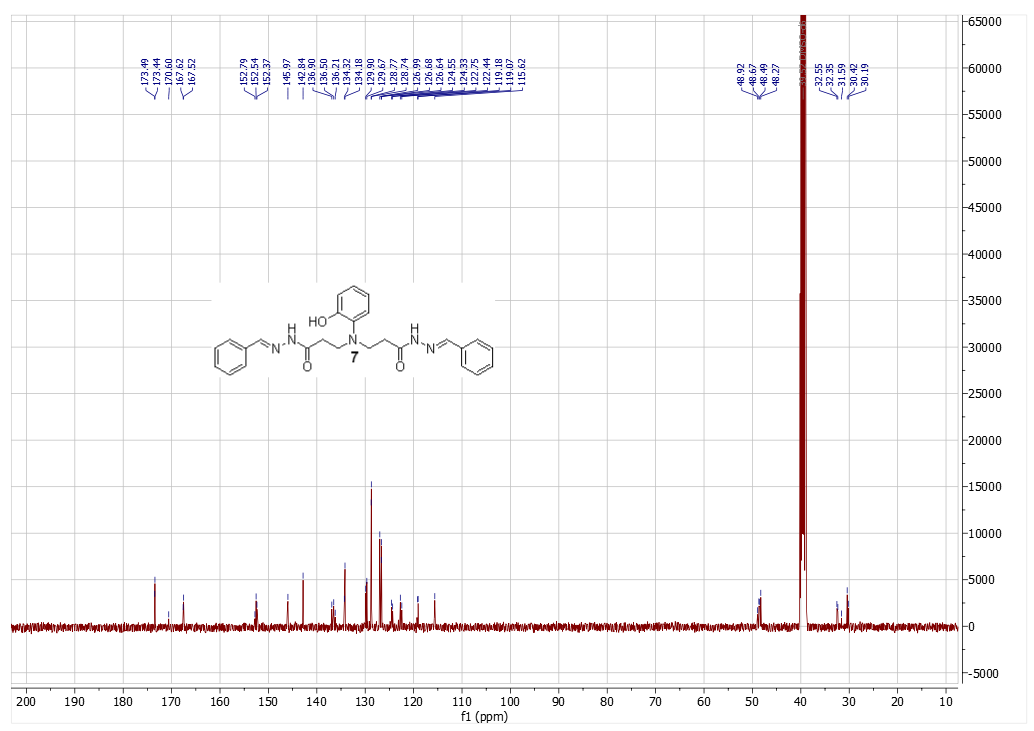
**

**Figure S12**. ^13^C NMR spectrum of compound **7**

*3,3'-((2-hydroxyphenyl)azanediyl)bis(N'-(2,4-difluorobenzylidene)propanehydrazide)* ***(8)***

*
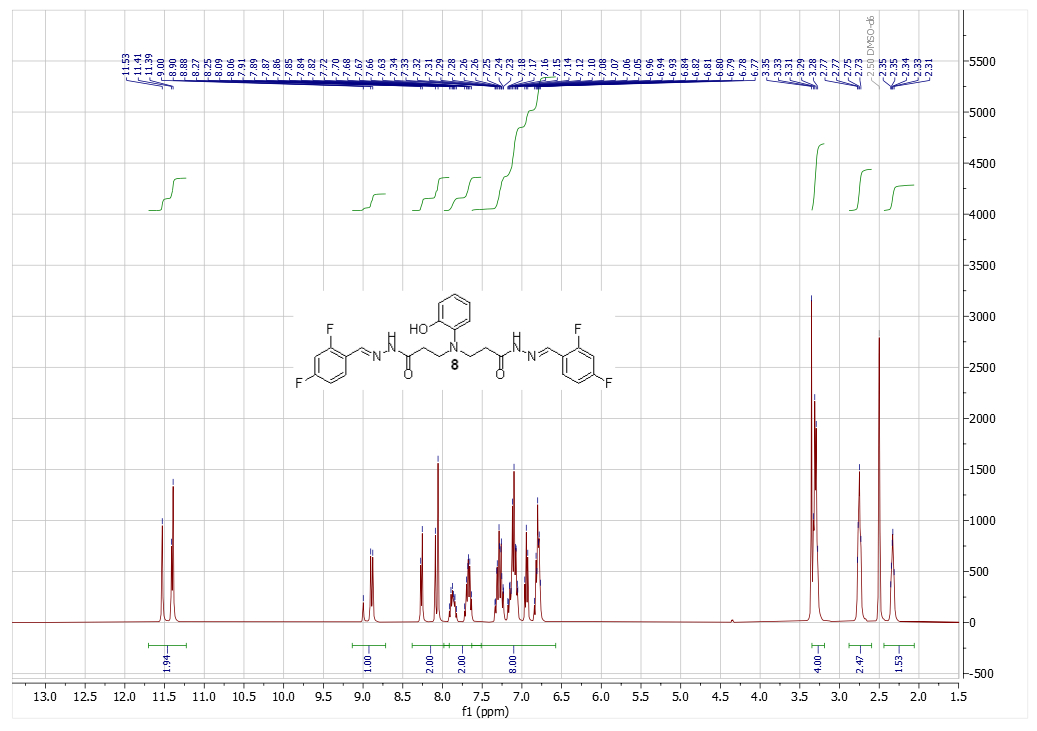
*

**Figure S13**. ^1^H NMR spectrum of compound **8**

**
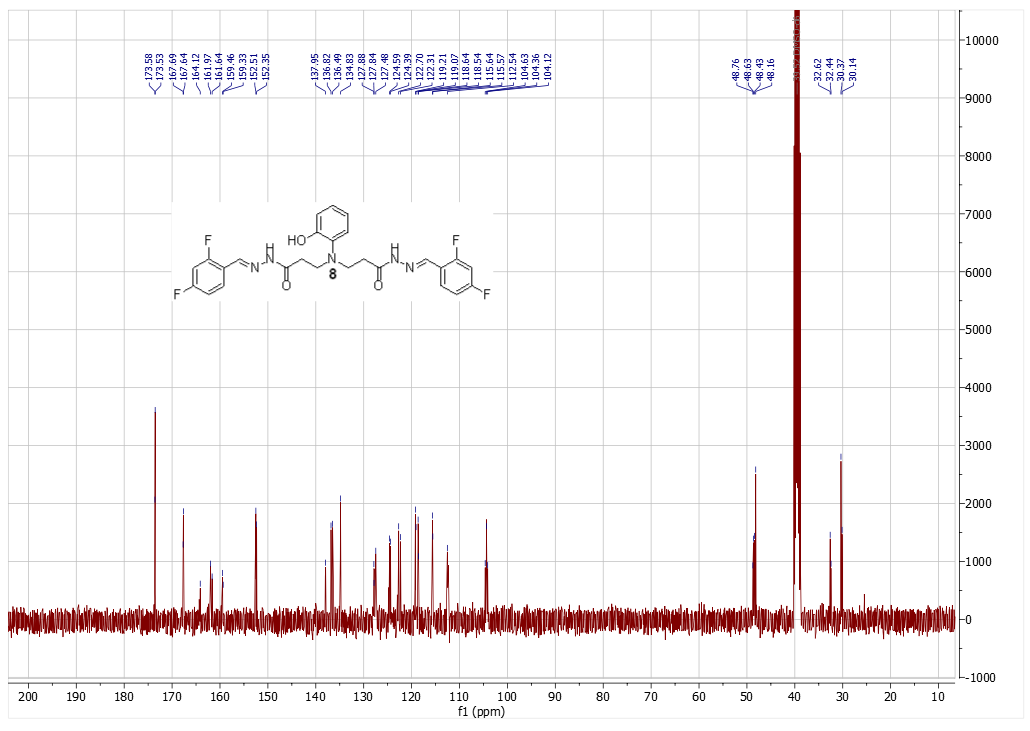
**

**Figure S14**. ^13^C NMR spectrum of compound **8**

*3,3'-((2-Hydroxyphenyl)azanediyl)bis(N'-(4-nitrobenzylidene)propanehydrazide)* ***(9)***

***
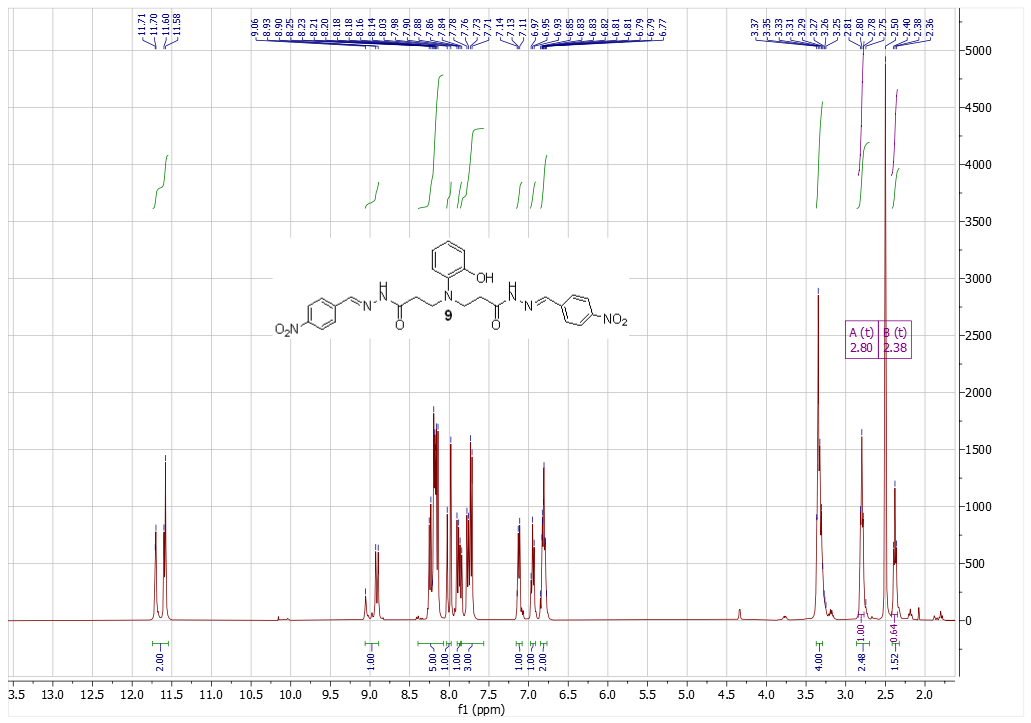
***

**Figure S15**. ^1^H NMR spectrum of compound **9**

**
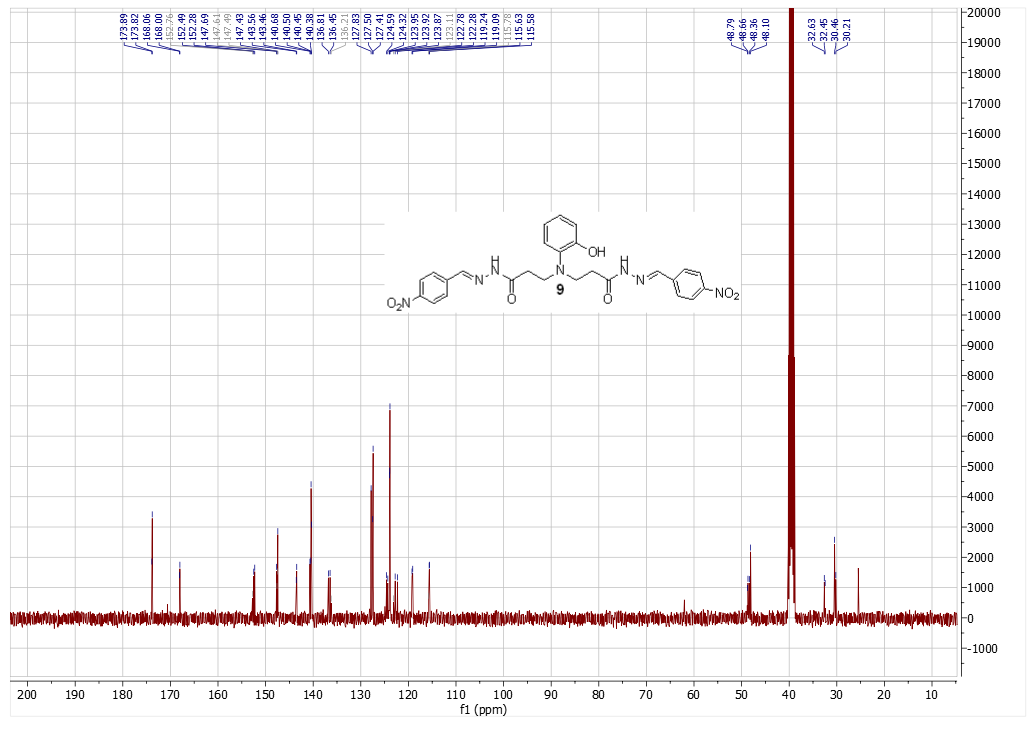
**

**Figure S16**. ^13^C NMR spectrum of compound **9**

*3,3'-((2-Hydroxyphenyl)azanediyl)bis(N'-(4-chlorobenzylidene)propanehydrazide)* ***(10)***

*
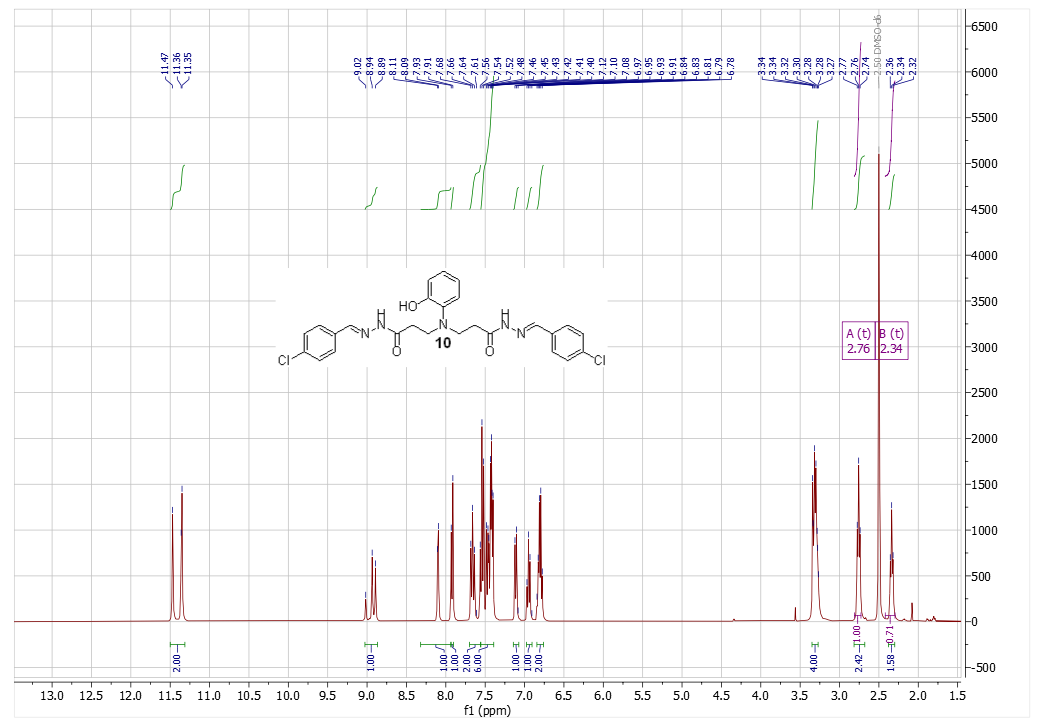
*

**Figure S17**. ^1^H NMR spectrum of compound **10**

**
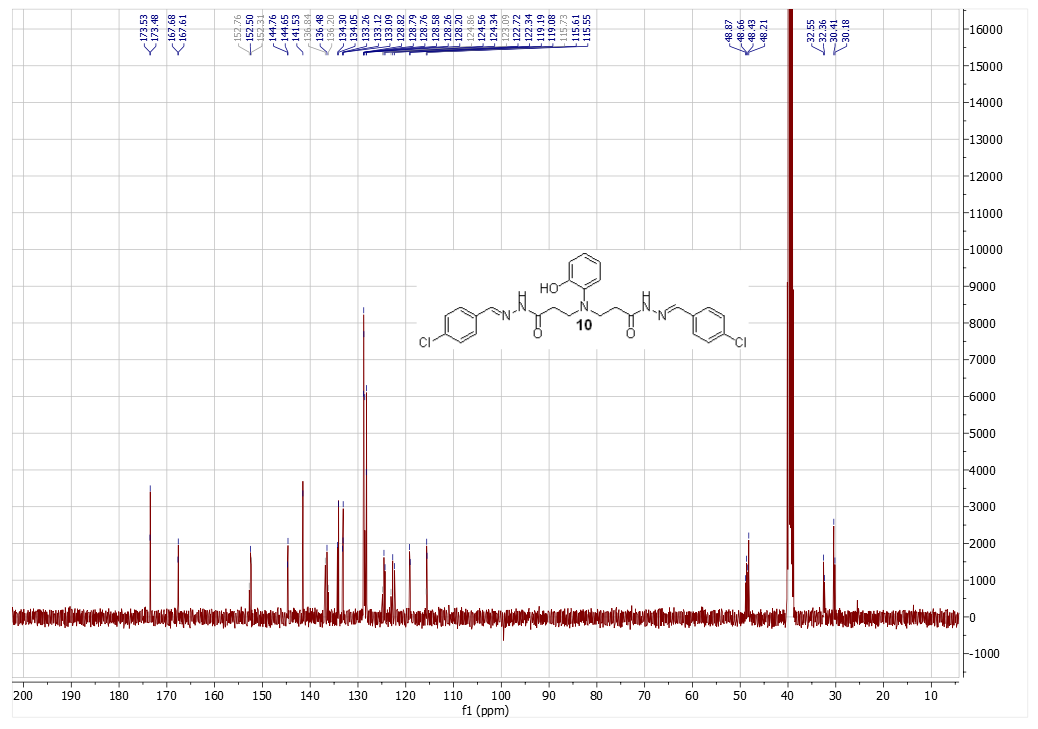
**

**Figure S18**. ^13^C NMR spectrum of compound **10**

*3,3'-((2-hydroxyphenyl)azanediyl)bis(N'-(4-(dimethylamino)benzylidene)propanehydrazide)* ***(11)***

*
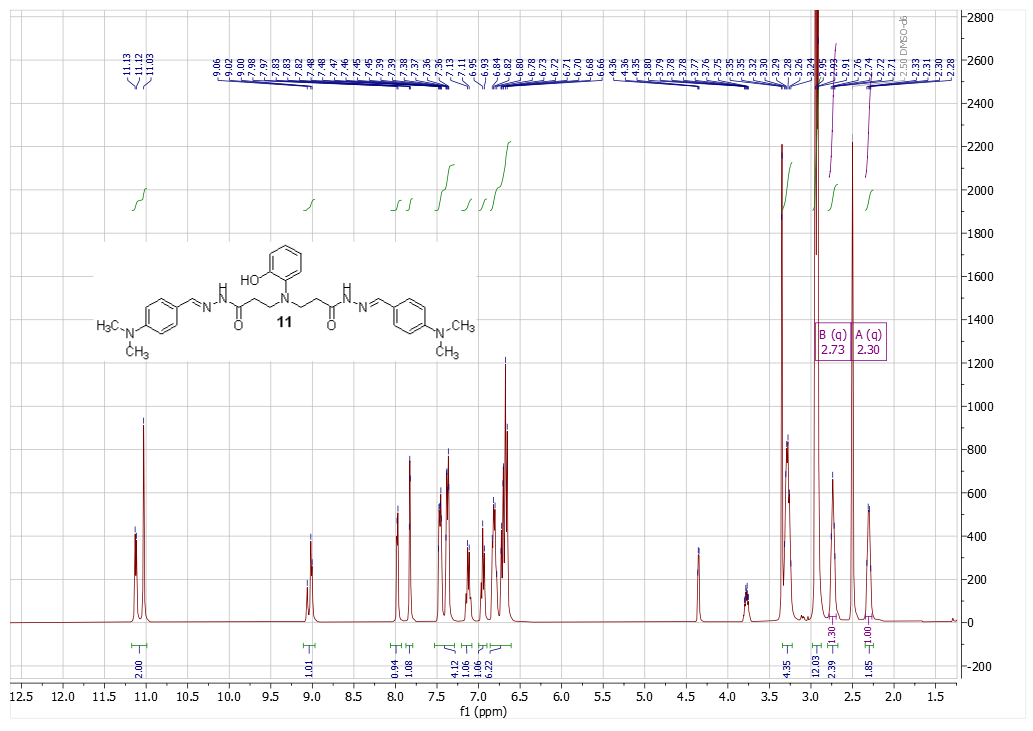
*

**Figure S19**. ^1^H NMR spectrum of compound **11**

**
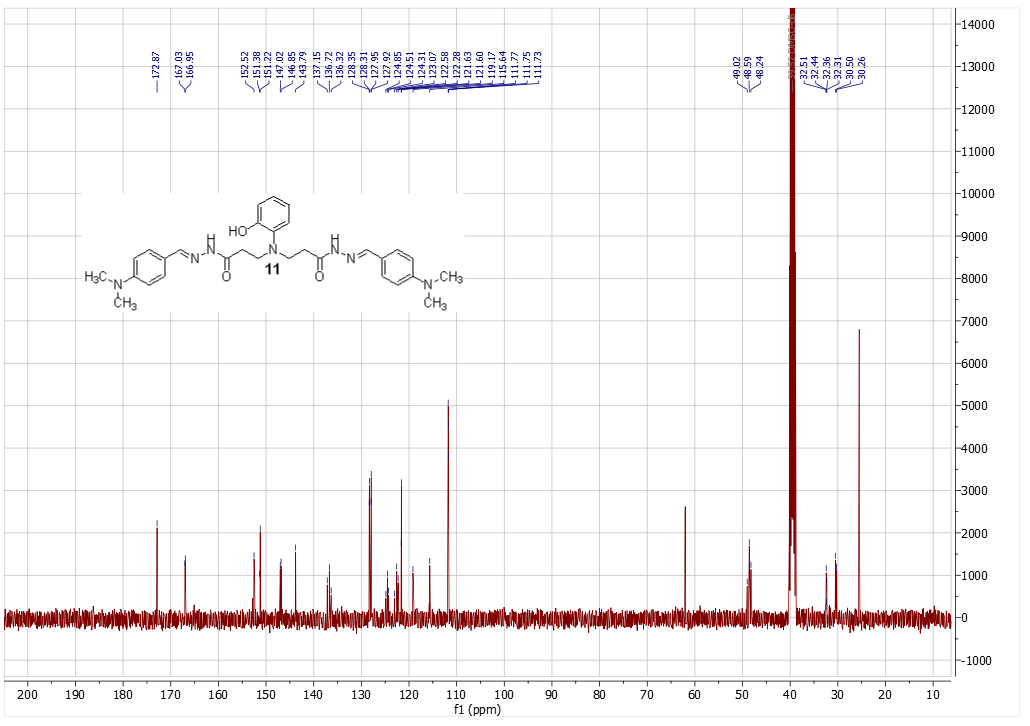
**

**Figure S20**. ^13^C NMR spectrum of compound **11**

*3,3'-((2-Hydroxyphenyl)azanediyl)bis(N'-(4-hydroxybenzylidene)propanehydrazide)* ***(12)***

*
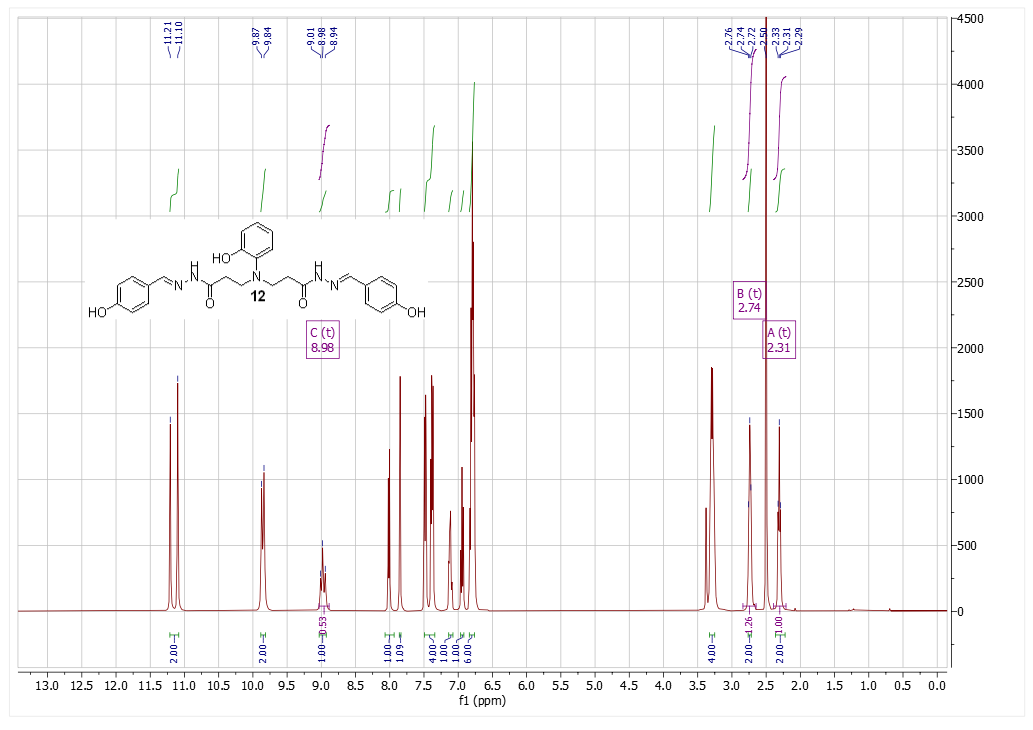
*

**Figure S21**. ^1^H NMR spectrum of compound **12**

**
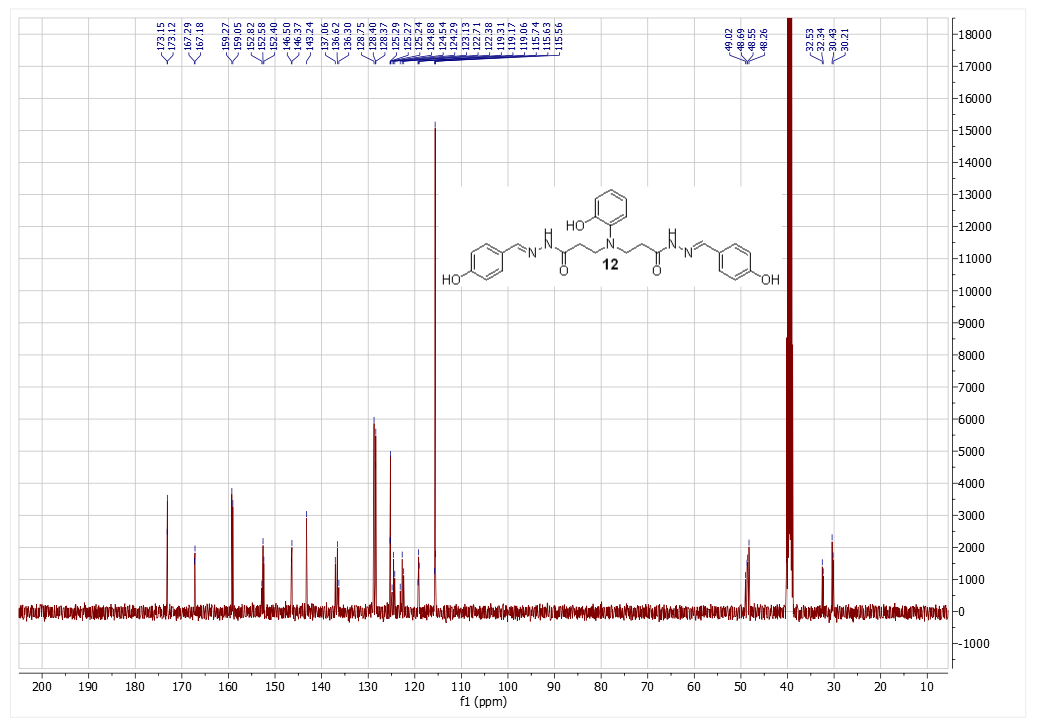
**

**Figure S22**. ^13^C NMR spectrum of compound **12**

*3,3'-((2-hydroxyphenyl)azanediyl)bis(N'-(3,4,5-trimethoxybenzylidene)propanehydrazide)* ***(13)***

***
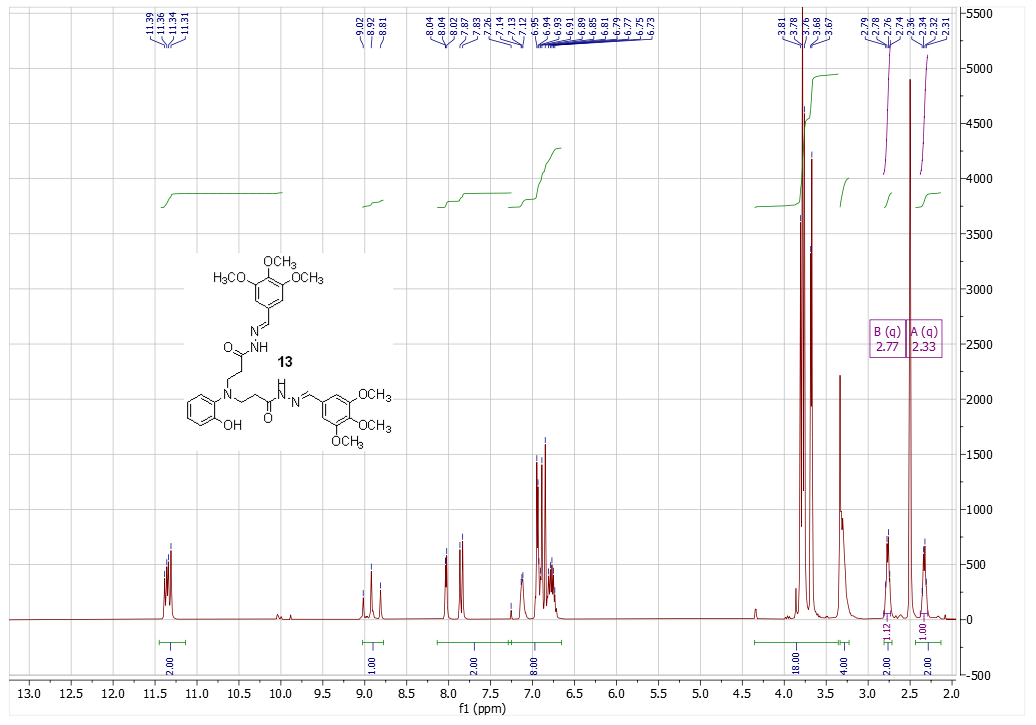
***

**Figure S23**. ^1^H NMR spectrum of compound **13**

**
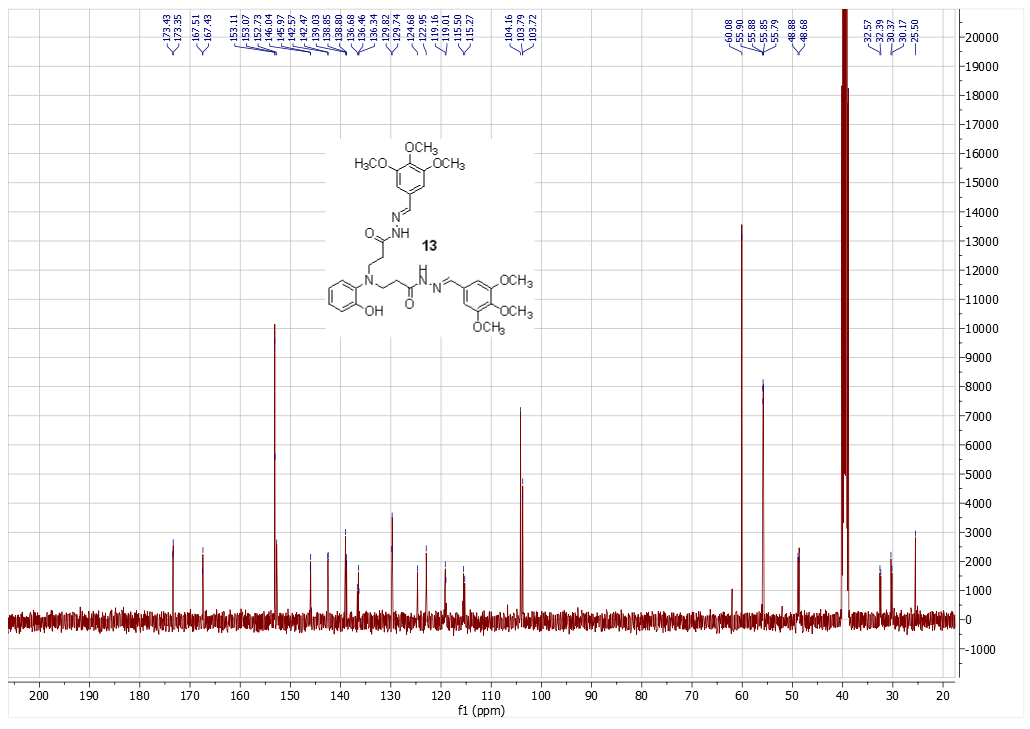
**

**Figure S24**. ^13^C NMR spectrum of compound **13**

*3,3'-((2-hydroxyphenyl)azanediyl)bis(N'-(naphthalen-1-ylmethylene)propanehydrazide)* ***(14)***

*
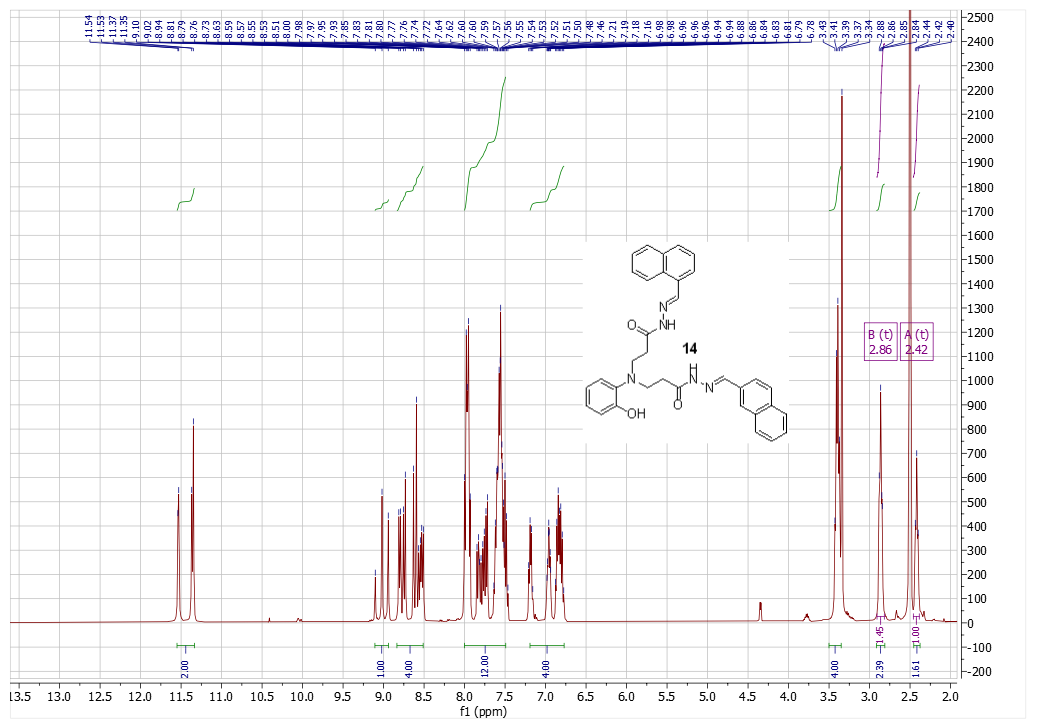
*

**Figure S25**. ^1^H NMR spectrum of compound **14**

**
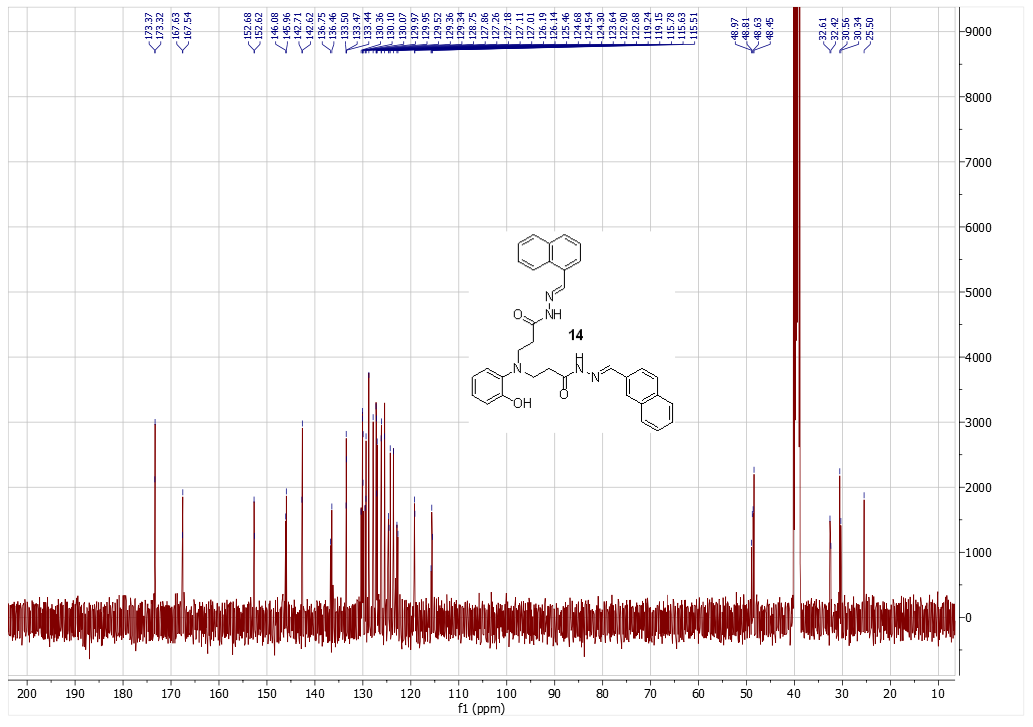
**

**Figure S26**. ^13^C NMR spectrum of compound **14**

*3,3'-((2-Hydroxyphenyl)azanediyl)bis(N'-(furan-2-ylmethylene)propanehydrazide)* ***(15)***

***
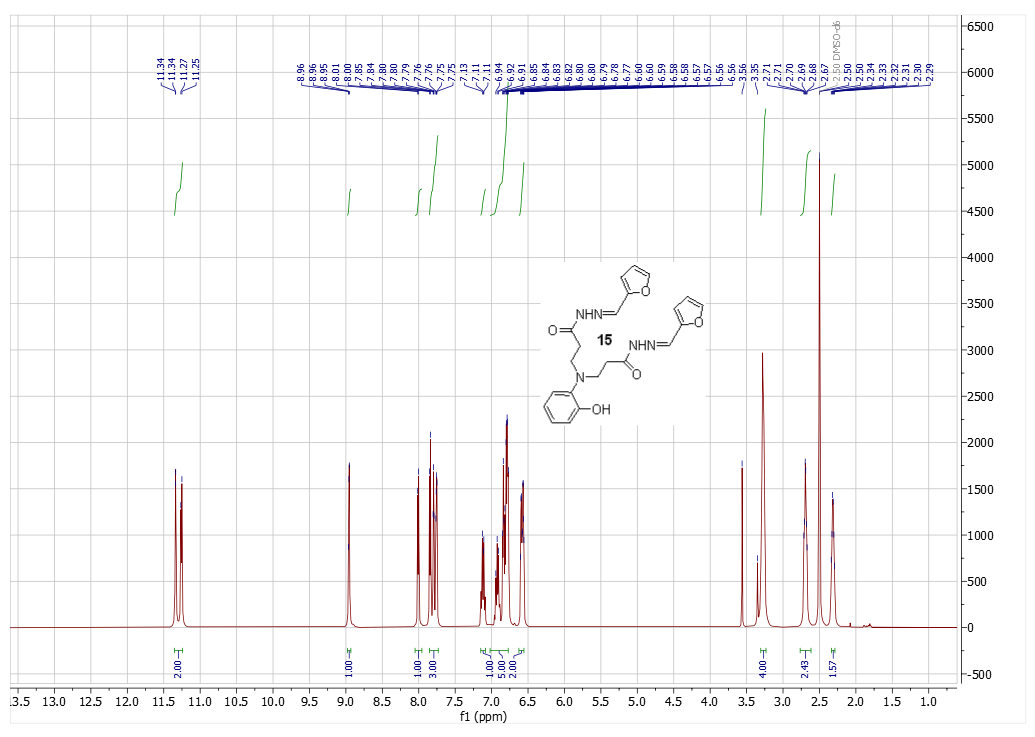
***

**Figure S26**. ^1^H NMR spectrum of compound **15**

**
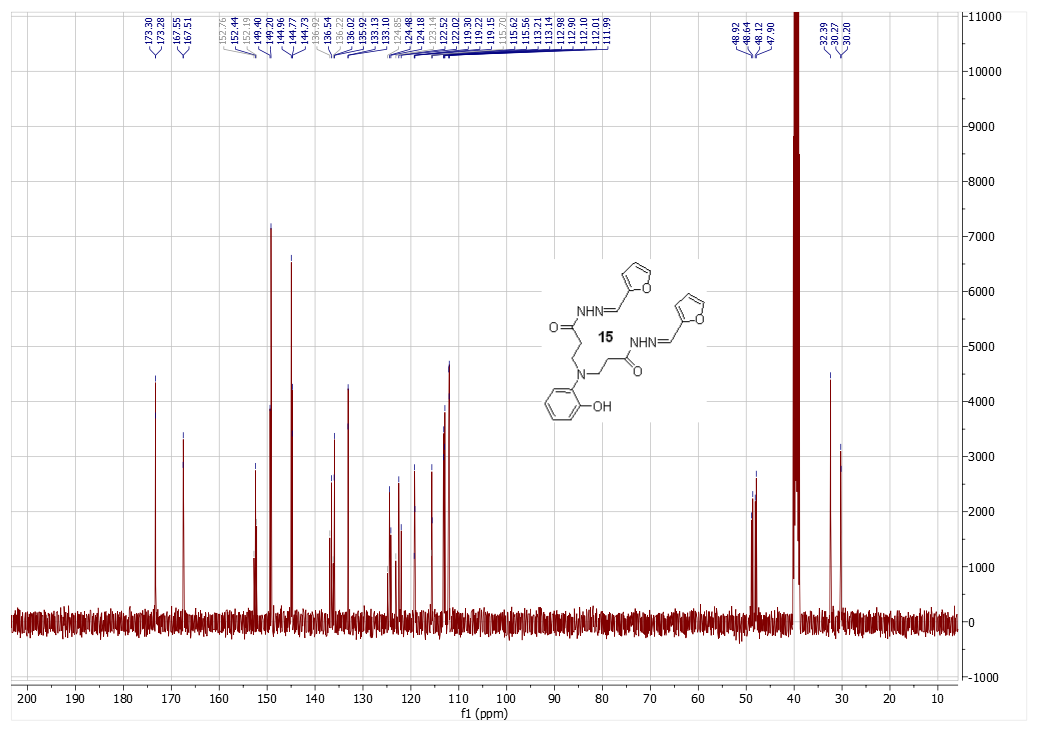
**

**Figure S27**. ^13^C NMR spectrum of compound **15**

*3,3'-((2-Hydroxyphenyl)azanediyl)bis(N'-(thiophen-2-ylmethylene)propanehydrazide)* ***(16)***

*
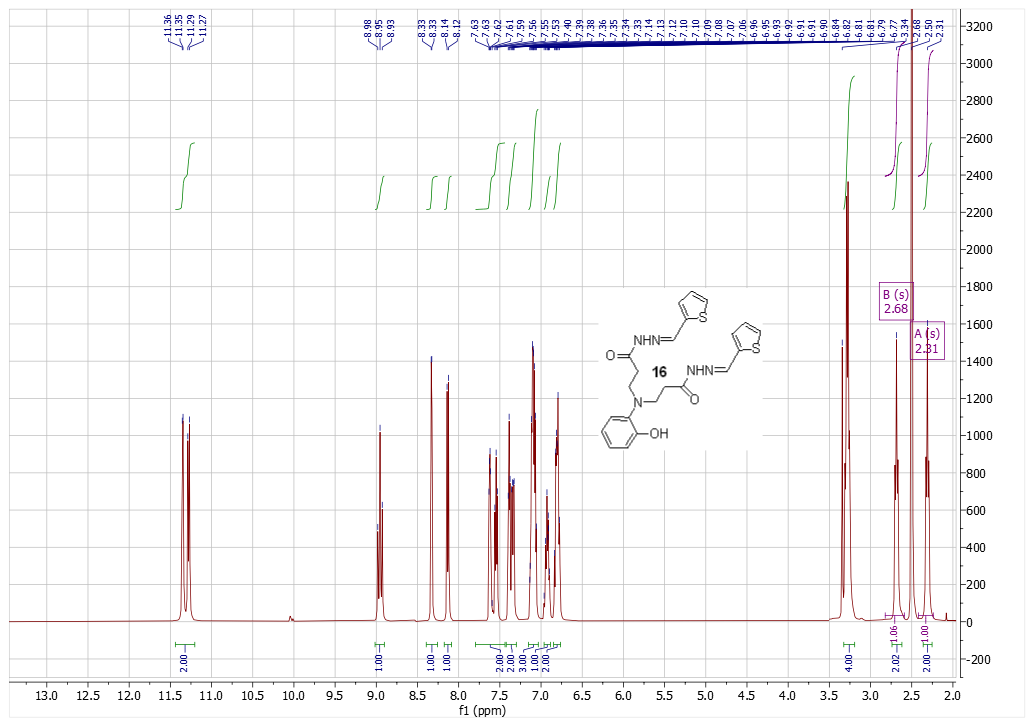
*

**Figure S28**. ^1^H NMR spectrum of compound **16**

**
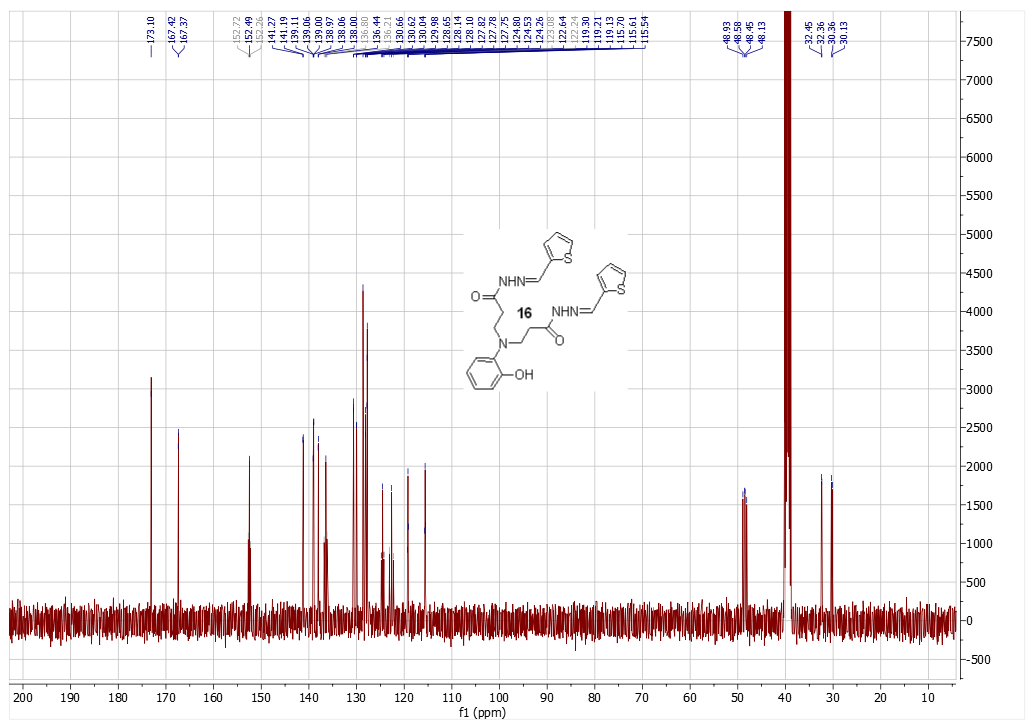
**

**Figure S29**. ^13^C NMR spectrum of compound **16**

*3,3'-((2-hydroxyphenyl)azanediyl)bis(N'-((5-nitrothiophen-2-yl)methylene)propanehydrazide)* ***(17)***

***
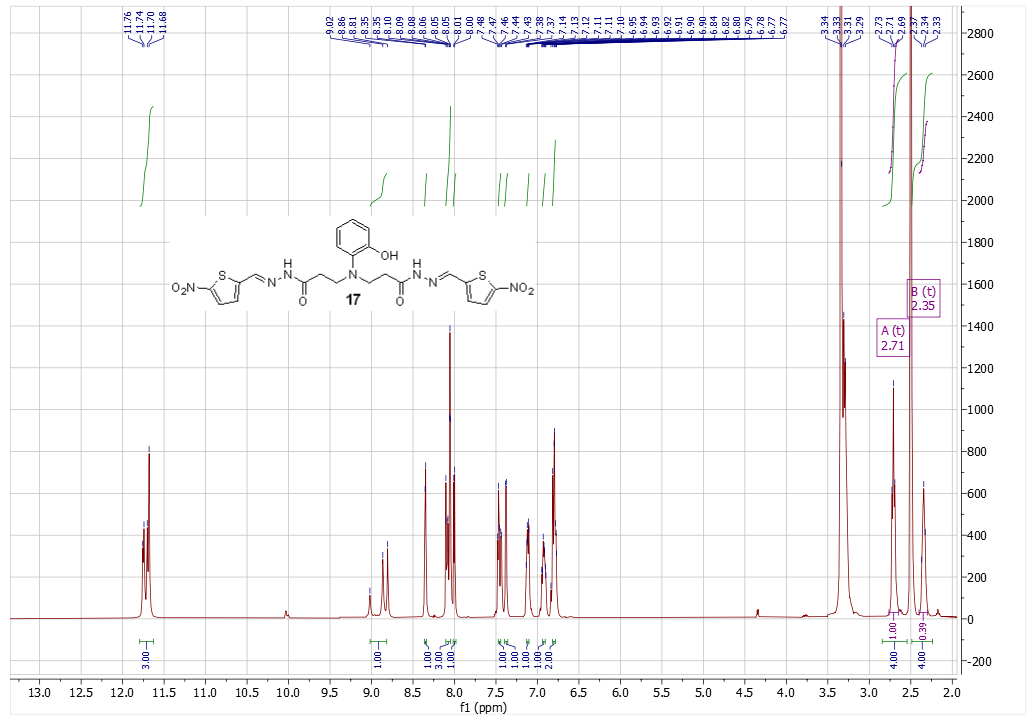
***

**Figure S30**. ^1^H NMR spectrum of compound **17**

**
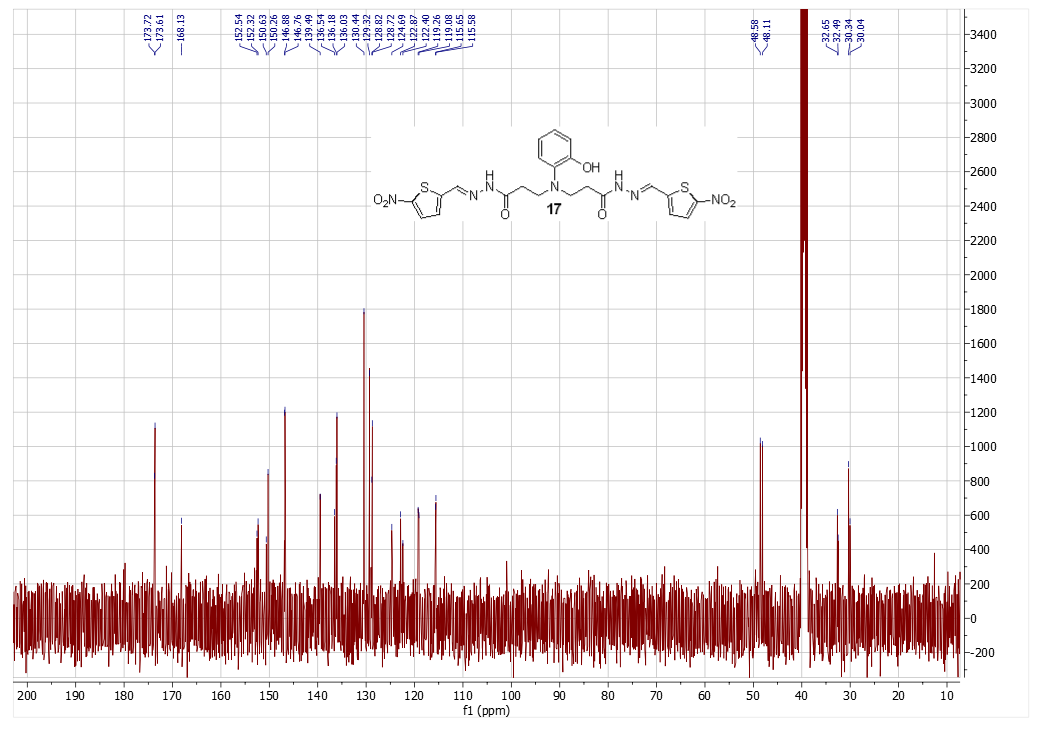
**

**Figure S31**. ^13^C NMR spectrum of compound **17**

*3,3'-((2-Hydroxyphenyl)azanediyl)bis(N'-(5-nitrofuran-2-ylmethylene)propanehydrazide)* ***(18)***

*
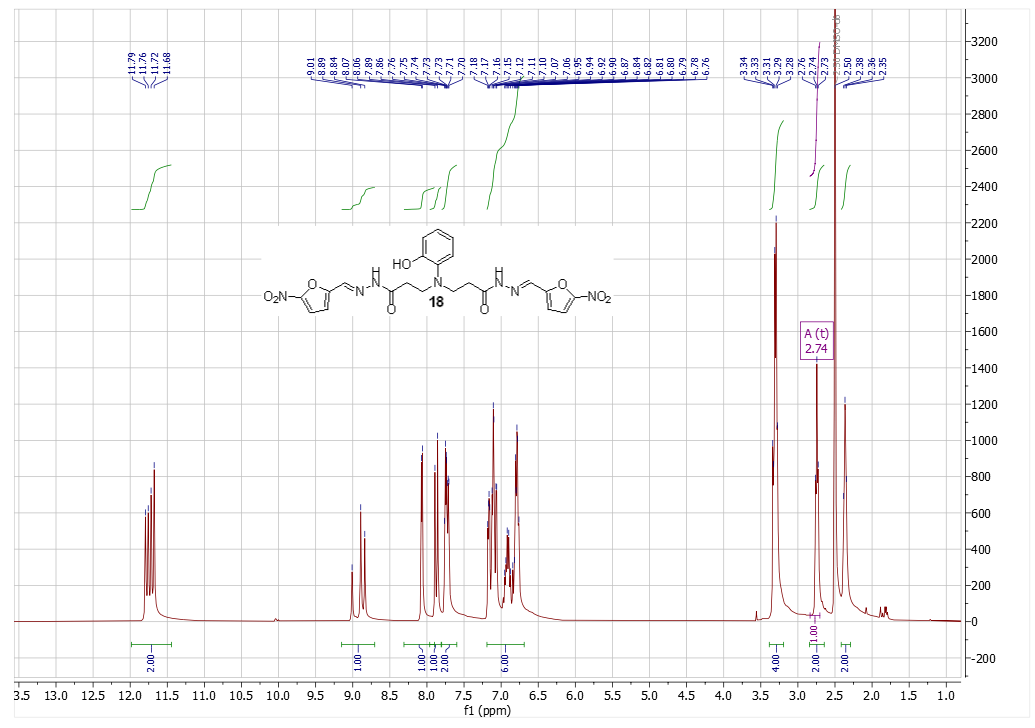
*

**Figure S32**. ^1^H NMR spectrum of compound **18**

**
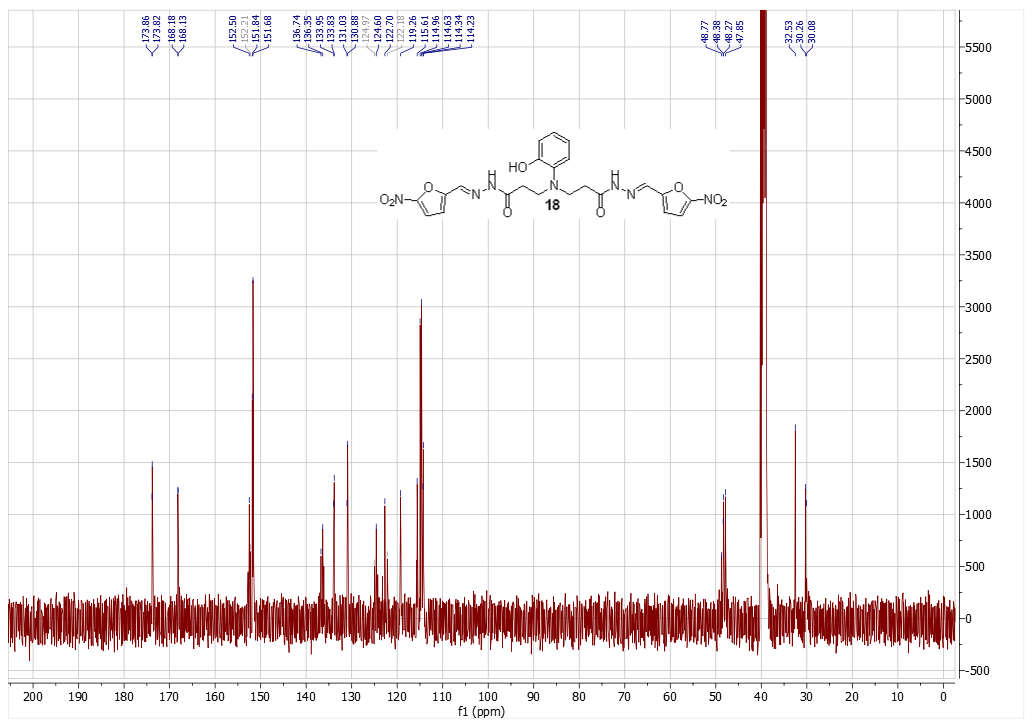
**

**Figure S33**. ^13^C NMR spectrum of compound **18**

*3,3'-((2-Hydroxyphenyl)azanediyl)bis(N'-(thiophen-3-ylmethylene)propanehydrazide)* ***(19)***

*
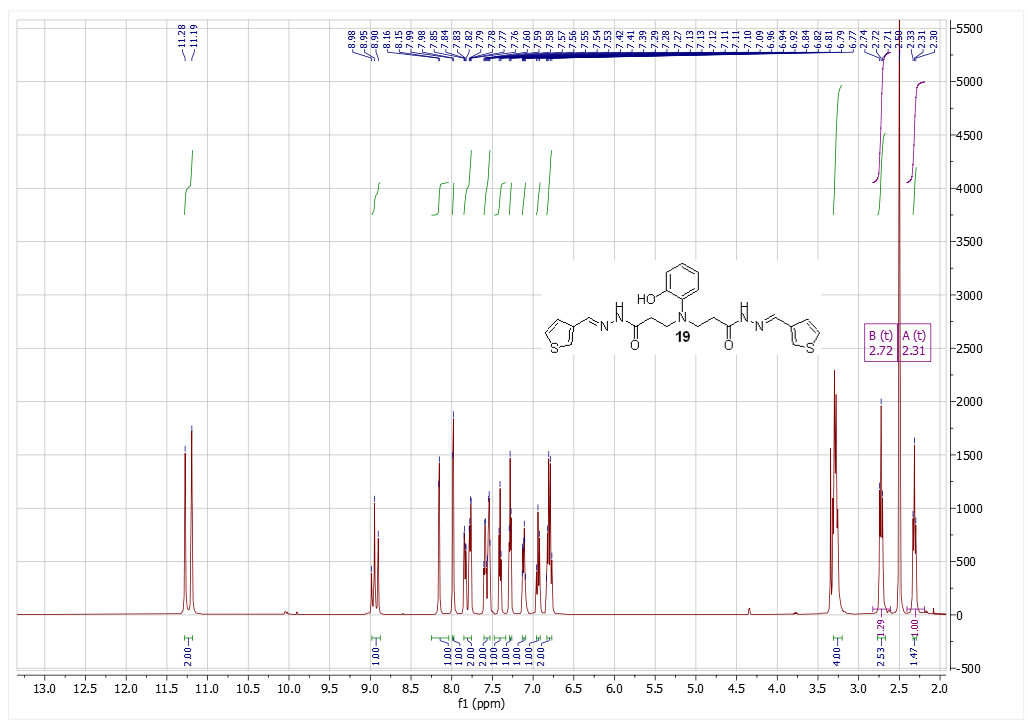
*

**Figure S34**. ^1^H NMR spectrum of compound **19**

**
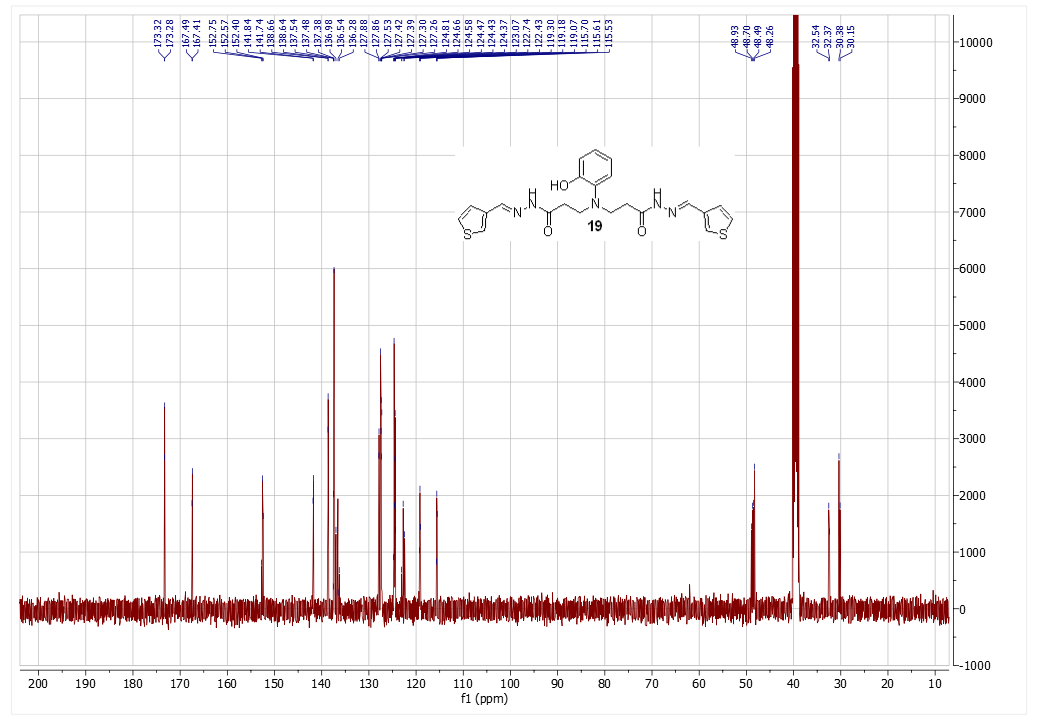
**

**Figure S35**. ^13^C NMR spectrum of compound **19**

*3,3'-((2-hydroxyphenyl)azanediyl)bis(N'-(propan-2-ylidene)propanehydrazide)* ***(20)***

*
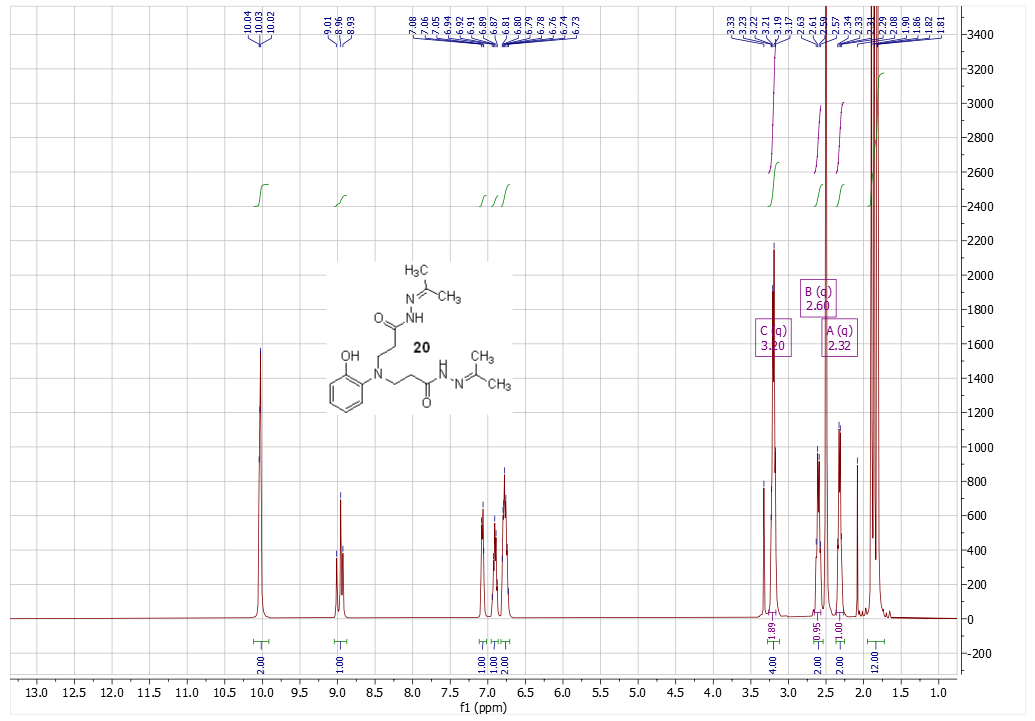
*

**Figure S36**. ^1^H NMR spectrum of compound **20**

**
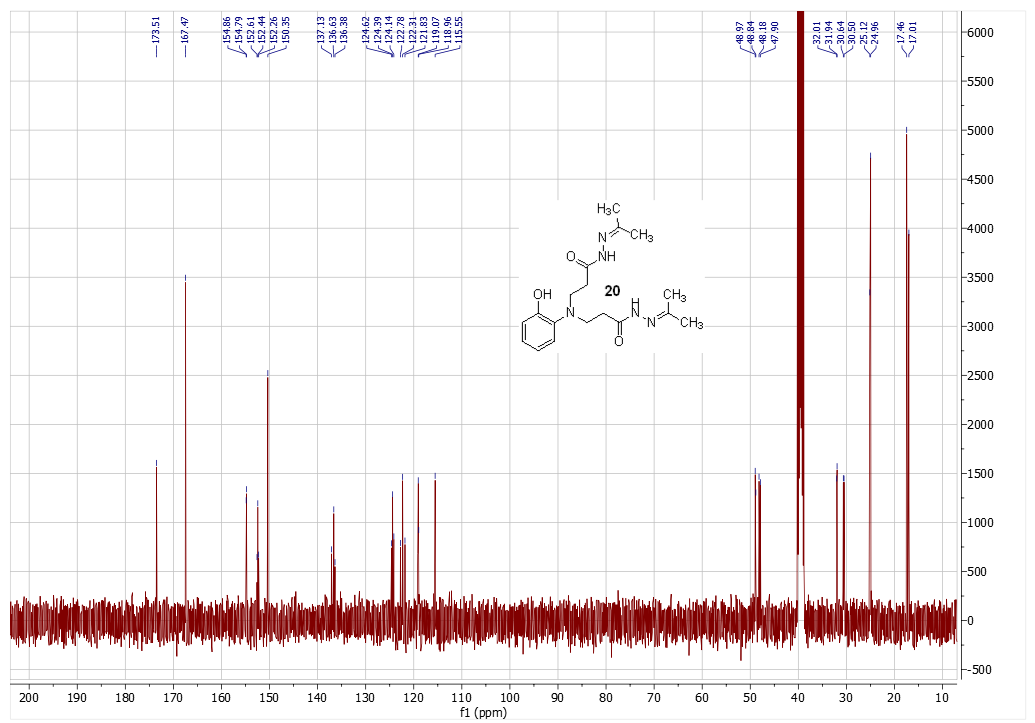
**

**Figure S37**. ^13^C NMR spectrum of compound **20**

*3,3'-((2-hydroxyphenyl)azanediyl)bis(N'-(butan-2-ylidene)propanehydrazide)* ***(21)***

***
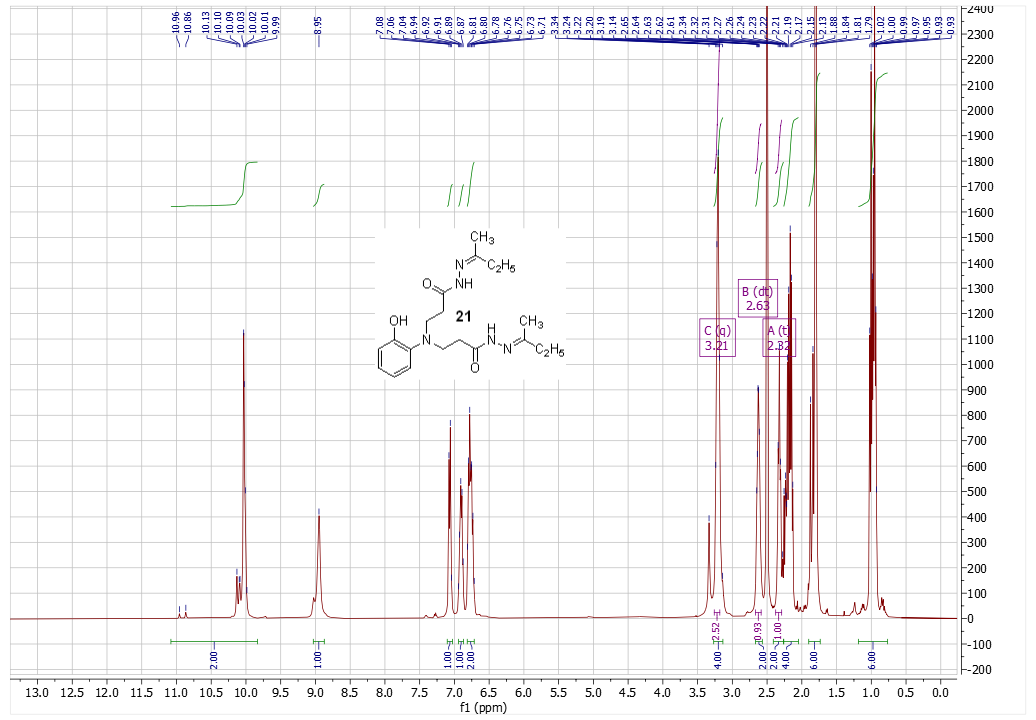
***

**Figure S38**. ^1^H NMR spectrum of compound **21**

**
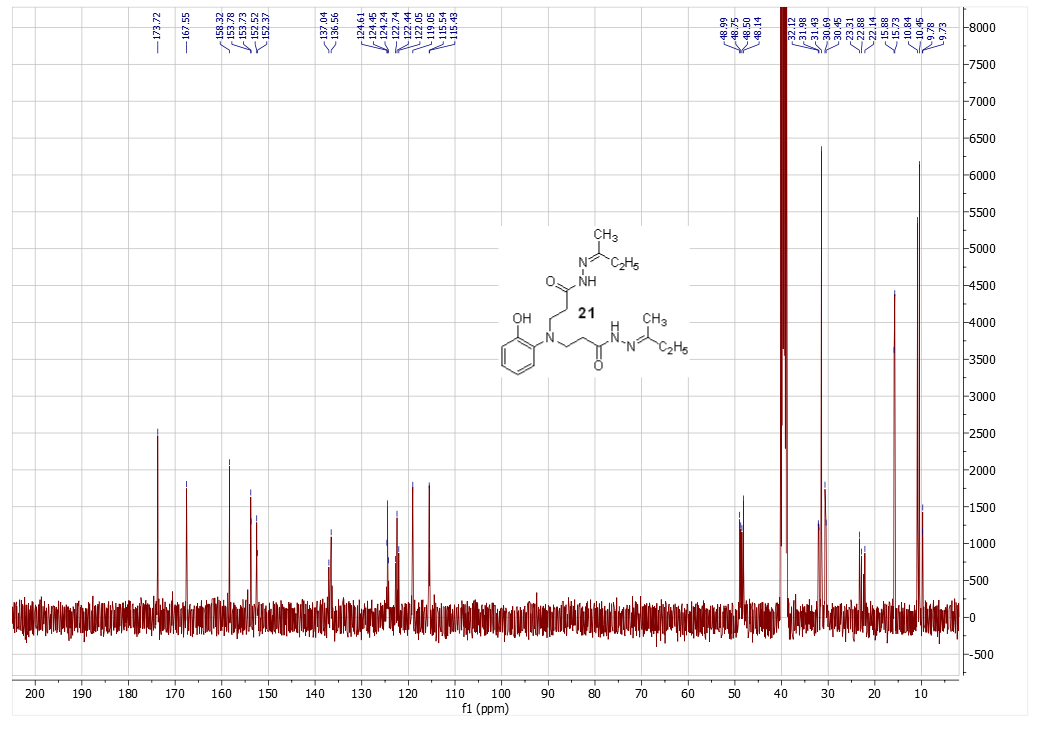
**

**Figure S39**. ^13^C NMR spectrum of compound **21**

*3,3'-((2-hydroxyphenyl)azanediyl)bis(N'-(1-phenylethylidene)propanehydrazide)* ***(22)***

***
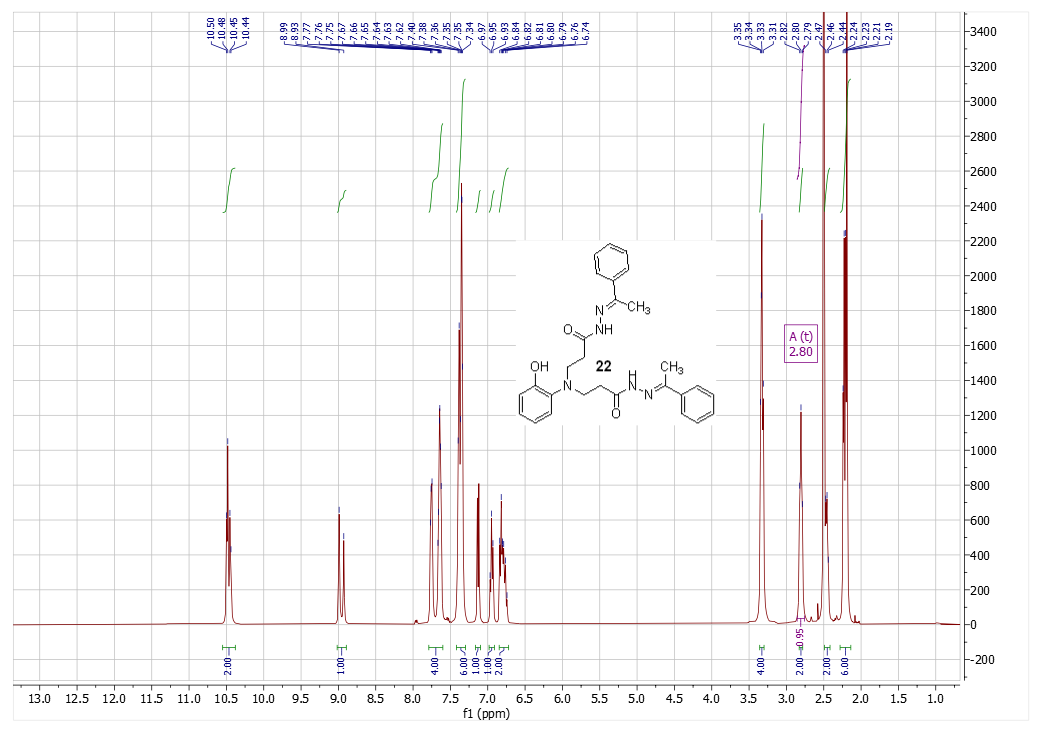
***

**Figure S40**. ^1^H NMR spectrum of compound **22**

**
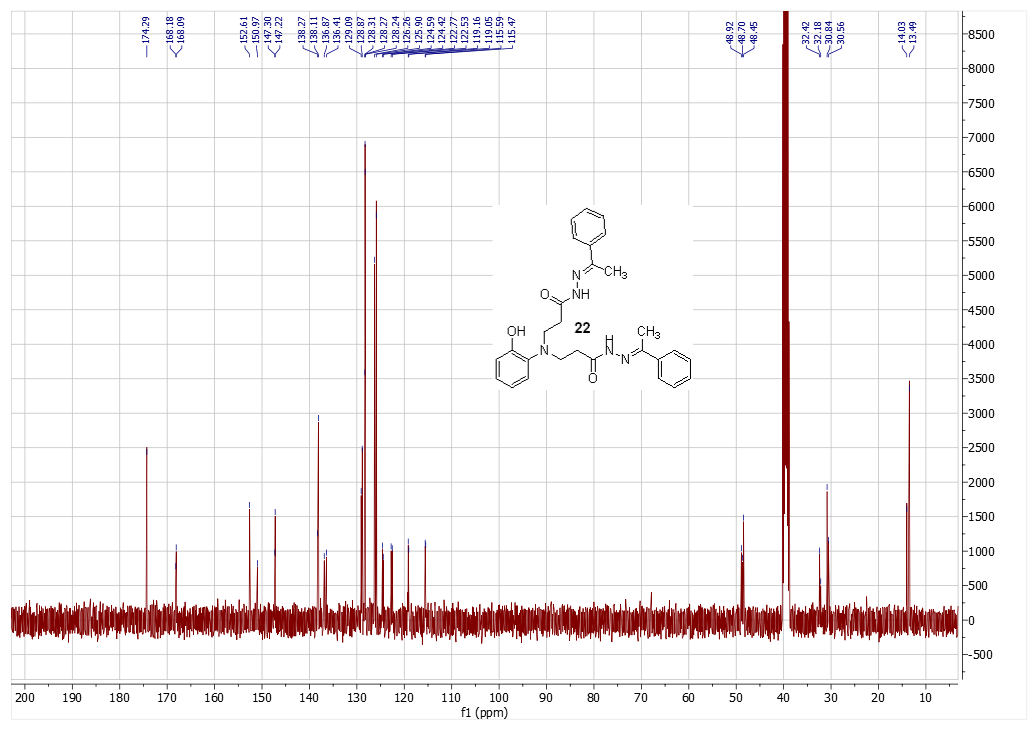
**

**Figure S41**. ^13^C NMR spectrum of compound **22**

*3,3'-((2-hydroxyphenyl)azanediyl)bis(N-(2,5-dimethyl-1H-pyrrol-1-yl)propanamide)* ***(23)***

*
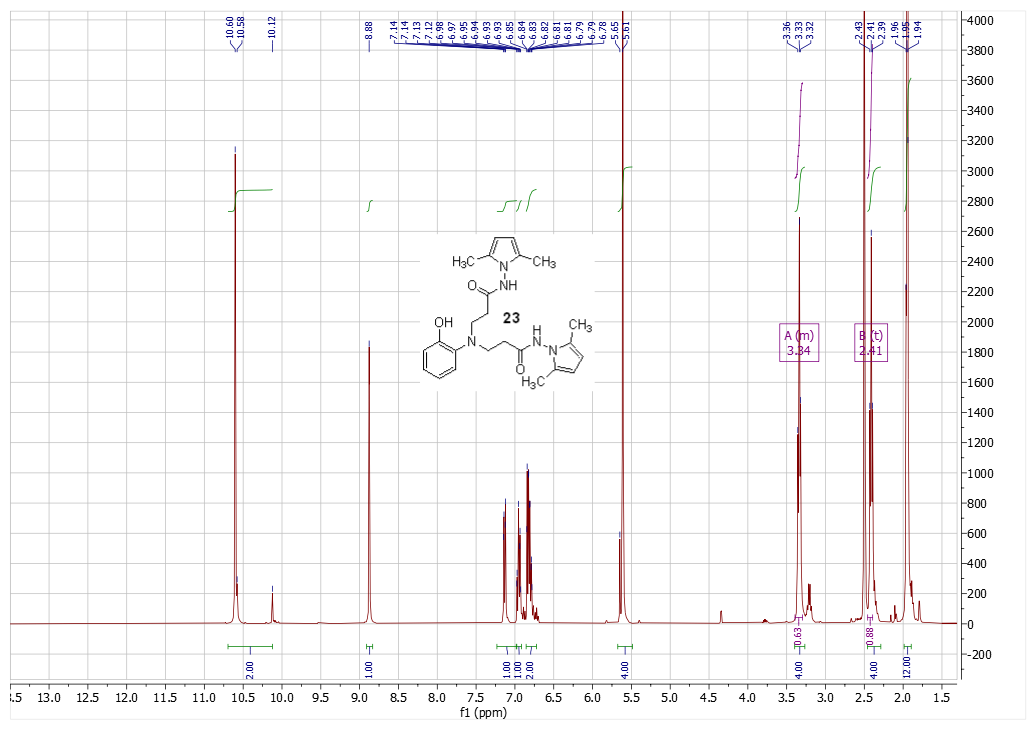
*

**Figure S42**. ^1^H NMR spectrum of compound **23**

**
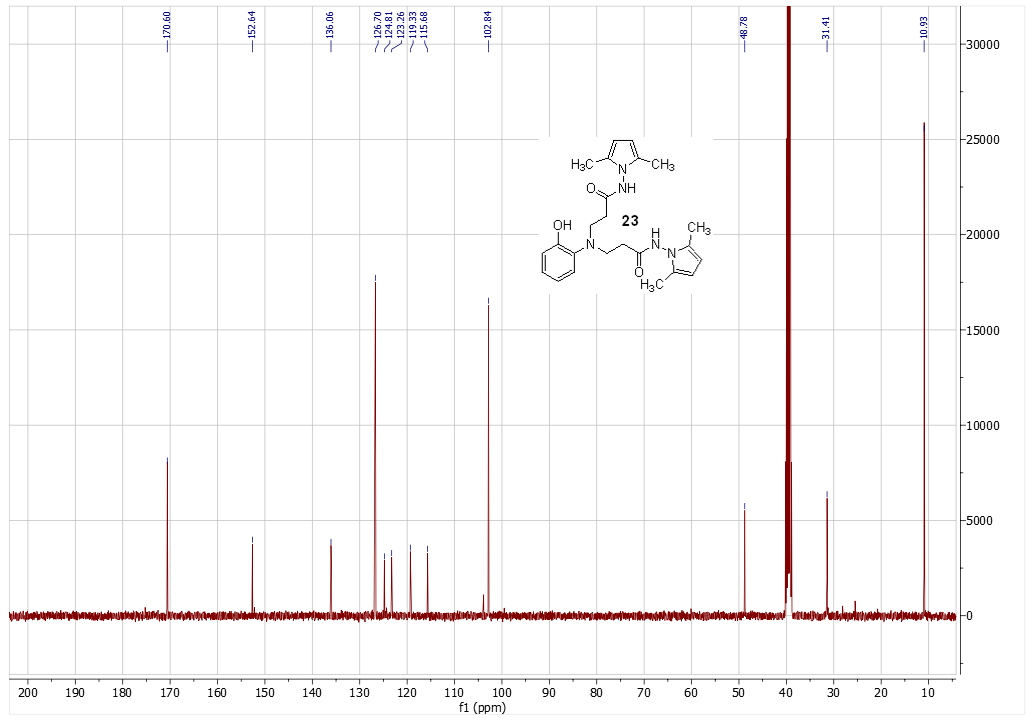
**

**Figure S43**. ^13^C NMR spectrum of compound **23**

*5-(3-(3,5-dimethyl-1H-pyrazol-1-yl)-3-oxopropyl)-4,5-dihydrobenzo[b][1,4]oxazepin-2(3H)-one* ***(24)***

*
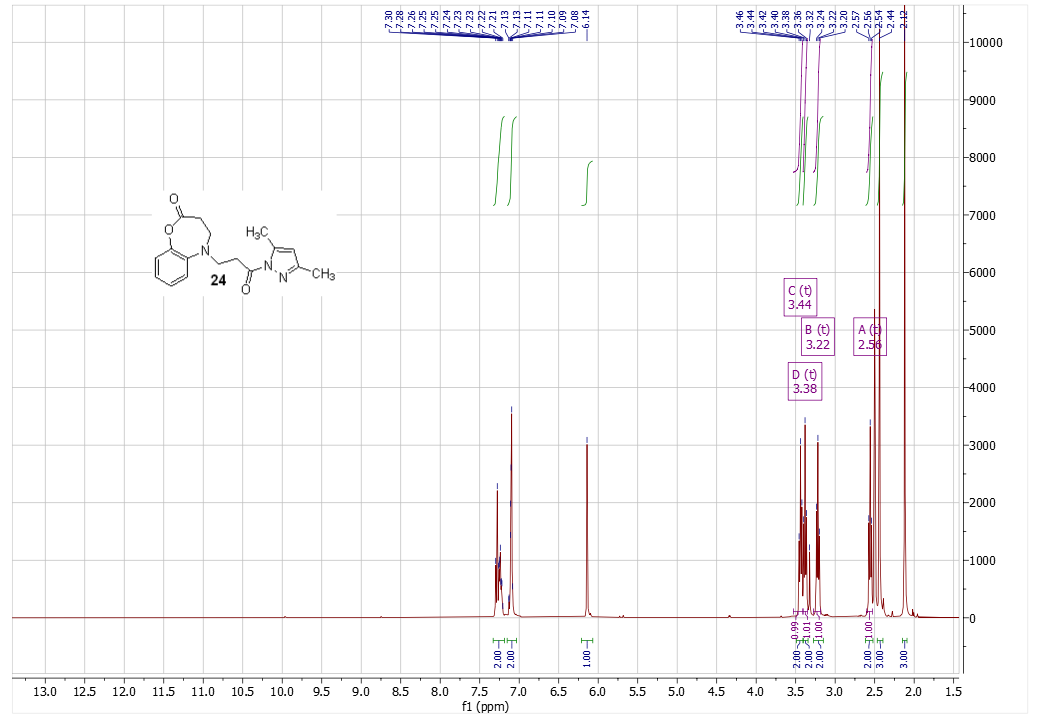
*

**Figure S44**. ^1^H NMR spectrum of compound **24**

**
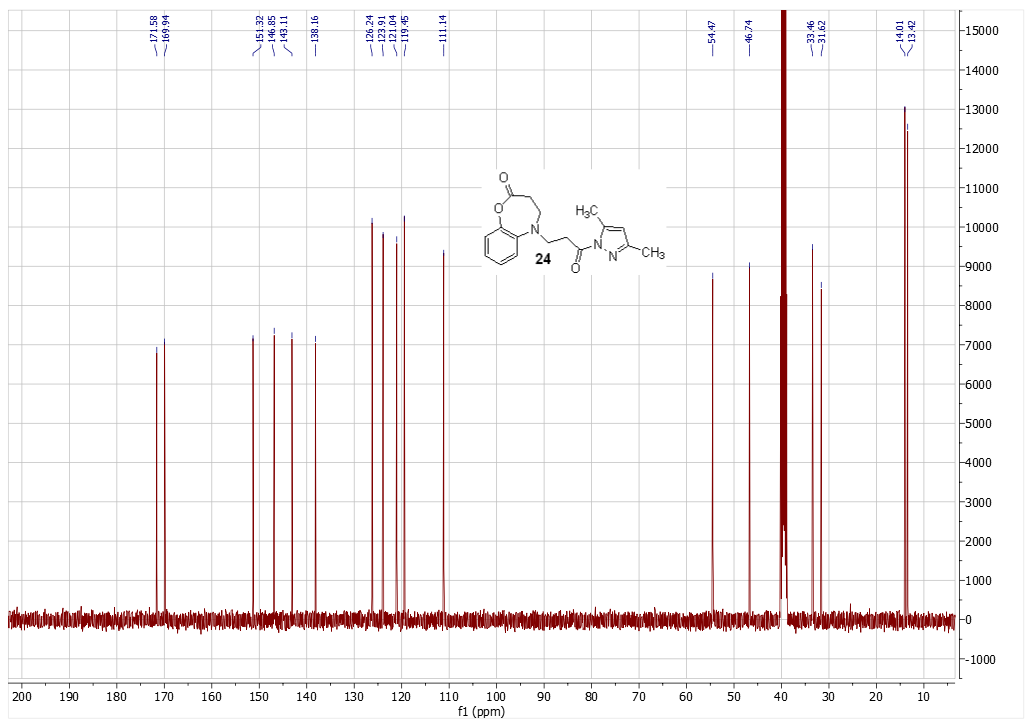
**

**Figure S45**. ^13^C NMR spectrum of compound **24**

*3,3'-((2-hydroxyphenyl)azanediyl)bis(N'-((Z)-2-oxoindolin-3-ylidene)propanehydrazide)* ***(25)***

*
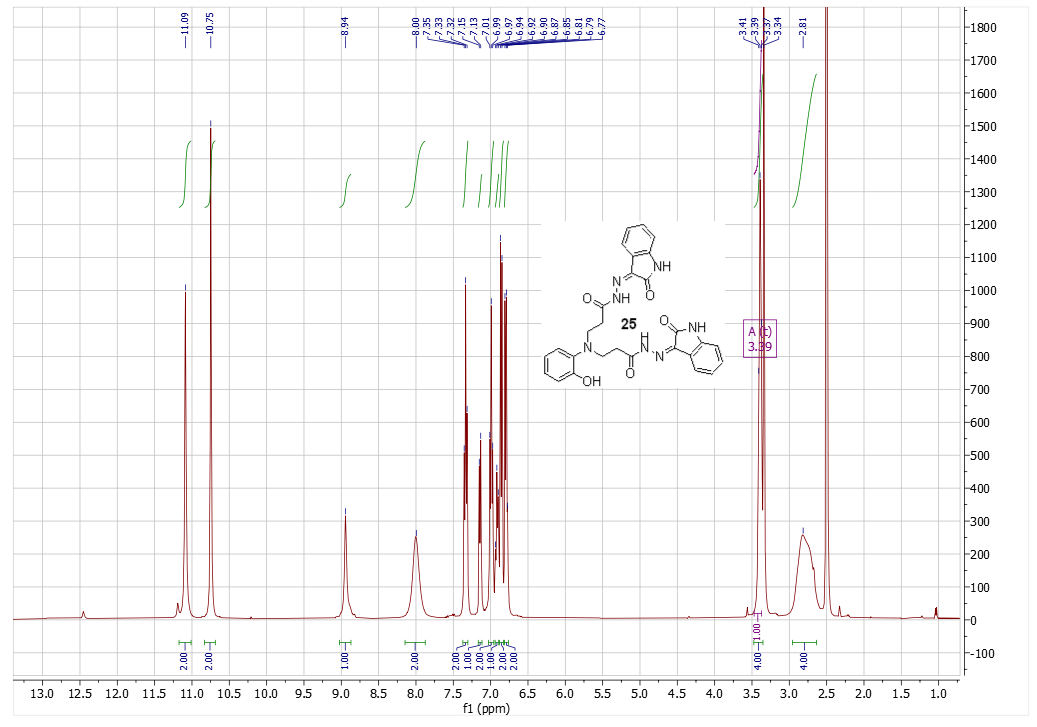
*

**Figure S46**. ^1^H NMR spectrum of compound **25**

**
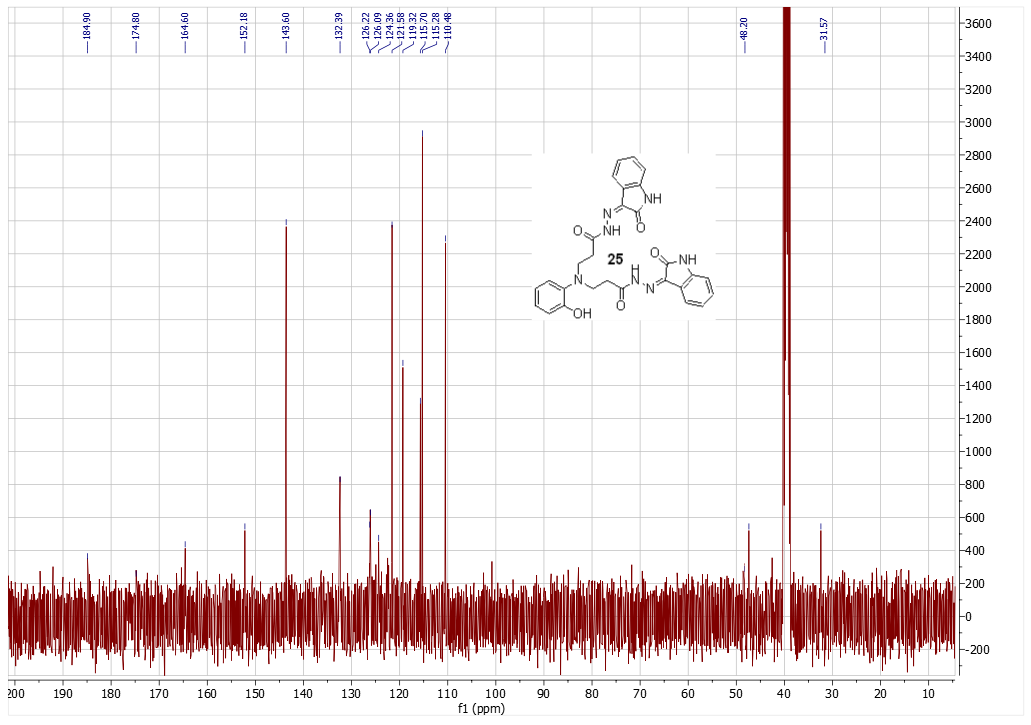
**

**Figure S47**. ^13^C NMR spectrum of compound **25**

*5,5'-(((2-hydroxyphenyl)azanediyl)bis(ethane-2,1-diyl))bis(1,3,4-oxadiazole-2(3H)-thione)* ***(26)***

***
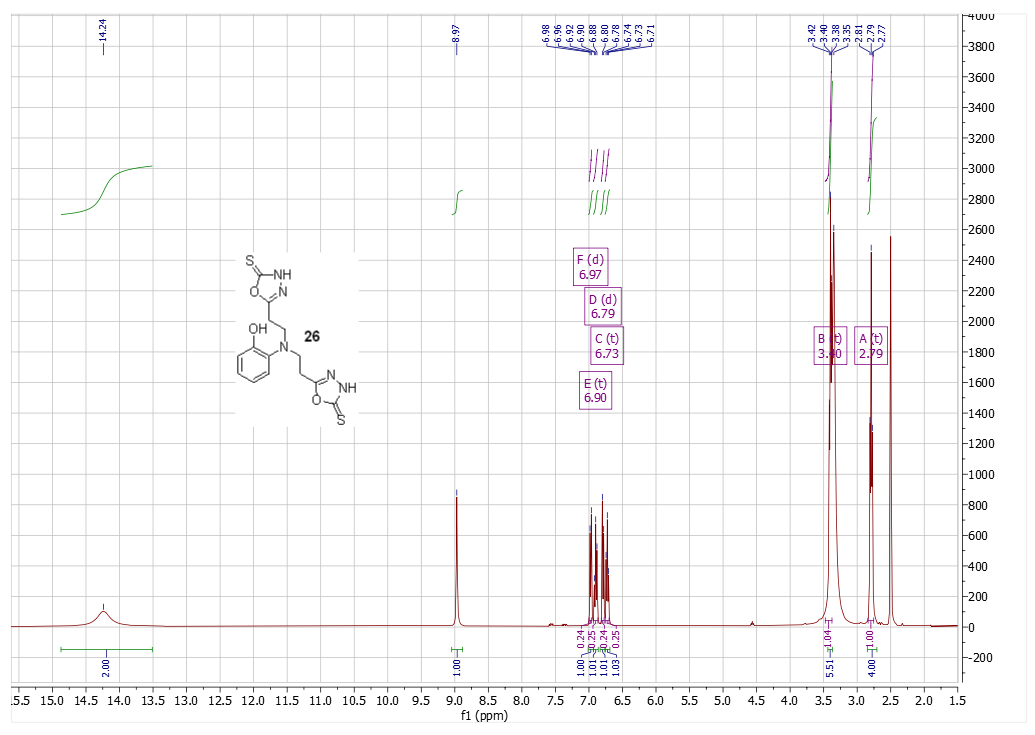
***

**Figure S48**. ^1^H NMR spectrum of compound **26**

**
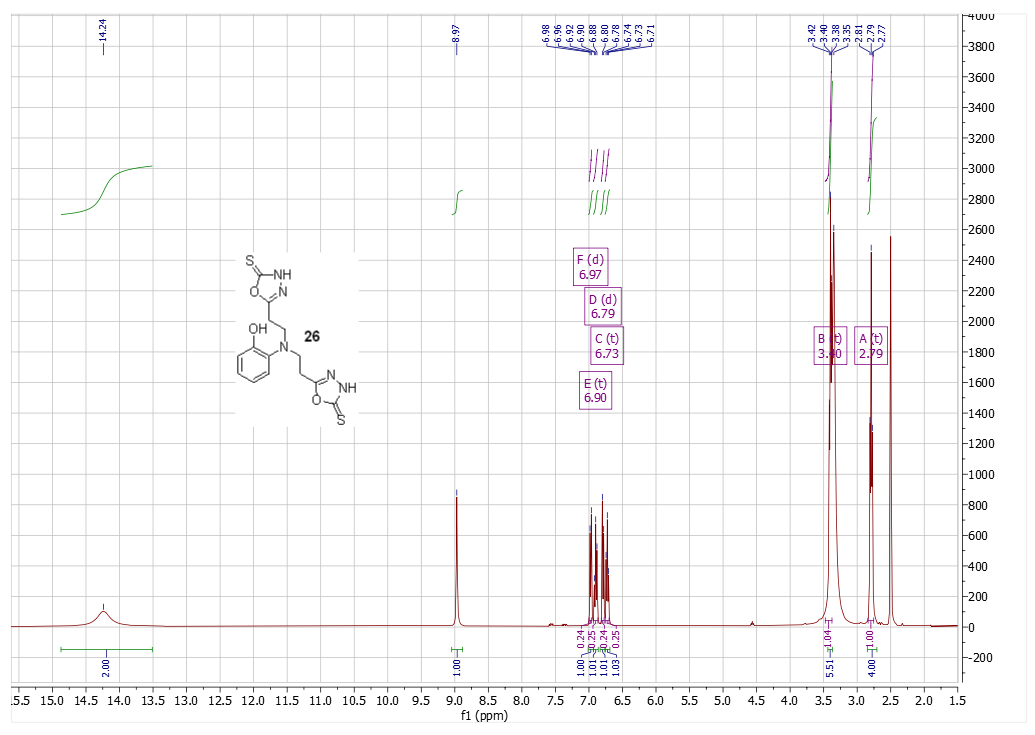
**

**Figure S49**. ^13^C NMR spectrum of compound **26**

*3,3'-((2-hydroxyphenyl)azanediyl)bis(N'-(2,4-difluorobenzylidene)propanehydrazide)* ***(8)***

**
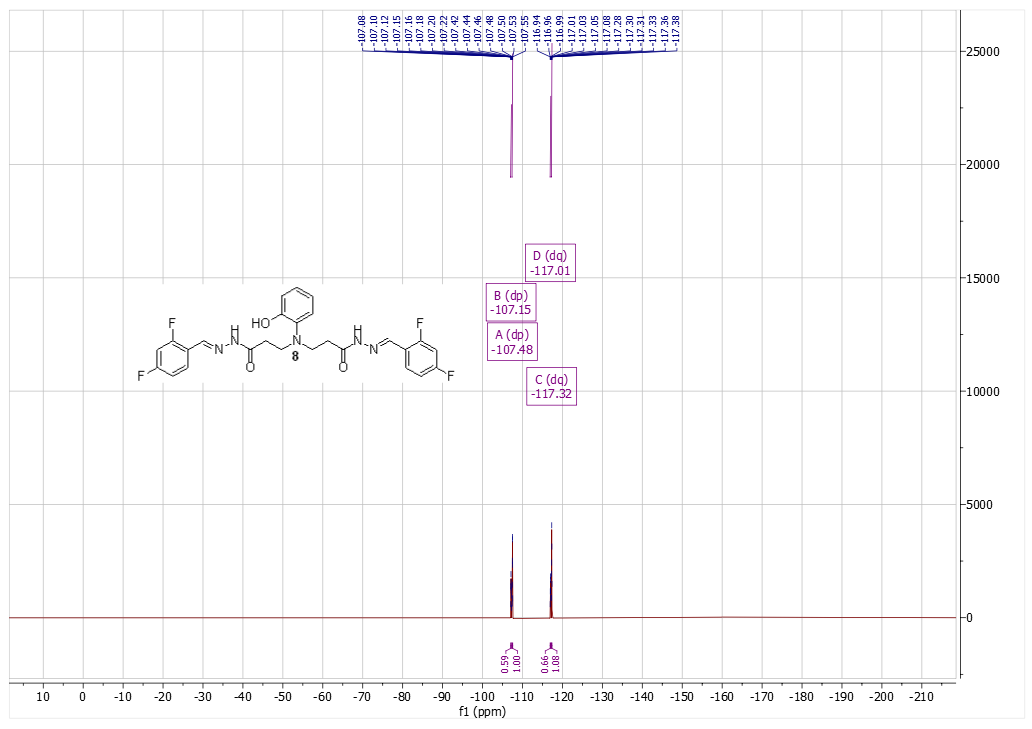
**

**Figure S50**. ^19^F NMR spectrum of compound **8**.

**Table S1.** The *in vitro* antimicrobial activity of *N*-substituted *β*-amino acid derivatives **2-26** against panel of fungal pathogens.

| **Compound** | **Minimal Inhibitory Concentration** | | | | | |
| --- | --- | --- | --- | --- | --- | --- |
|  | ***C. albicans*** | ***C. krusei*** | ***C. auris*** | ***A. fumigatus*** | ***R. delamar*** | ***C. bertholatiae*** |
| **2** | 128> | 128> | 128> | 128> | 128> | 128> |
| **3** | 128> | 128> | 128> | 128> | 128> | 128> |
| **4** | 128> | 128> | 128> | 128> | 128> | 128> |
| **5** | 128> | 128> | 128> | 128> | 128> | 128> |
| **6** | 128> | 128> | 128> | 128> | 128> | 128> |
| **7** | 128> | 128> | 128> | 128> | 128> | 128> |
| **8** | 128> | 128> | 128> | 128> | 128> | 128> |
| **9** | 128> | 128> | 128> | 128> | 128> | 128> |
| **10** | 128> | 128> | 128> | 128> | 128> | 128> |
| **11** | 128> | 128> | 128> | 128> | 128> | 128> |
| **12** | 128> | 128> | 128> | 128> | 128> | 128> |
| **13** | 128> | 128> | 128> | 128> | 128> | 128> |
| **14** | 128> | 128> | 128> | 128> | 128> | 128> |
| **15** | 128> | 128> | 128> | 128> | 128> | 128> |
| **16** | 128> | 128> | 128> | 128> | 128> | 128> |
| **17** | 128> | 128> | 128> | 128> | 128> | 128> |
| **18** | 128> | 128> | 128> | 128> | 128> | 128> |
| **19** | 128> | 128> | 128> | 128> | 128> | 128> |
| **20** | 128> | 128> | 128> | 128> | 128> | 128> |
| **21** | 128> | 128> | 128> | 128> | 128> | 128> |
| **22** | 128> | 128> | 128> | 128> | 128> | 128> |
| **23** | 128> | 128> | 128> | 128> | 128> | 128> |
| **24** | 128> | 128> | 128> | 128> | 128> | 128> |
| **25** | 128 | 128> | 128> | 128> | 128> | 128> |
| **26** | 16 | 128> | 128 | 128> | 128> | 128> |
| **Amphotericin B** | 0.5 | 1 | 2 | 1 | 0.5 | 2 |
| **Fluconazole** | 1 | 16 | 128 | 128> | 128> | 128> |
| **Posaconazole** | 1 | 2 | 8 | 8 | 0.5 | 1 |
| **Voriconazole** | 1 | 1 | 4 | 16 | 1 | 0.5 |

**Table S2.** Crystal data and structure refinement for compound **26** (named BGiii159).

| **Identification code** | B-G-3-159 |
| --- | --- |
| **Empirical formula** | C_17_H_19_N_3_O_3_ |
| **Formula weight** | 313.35 |
| **Temperature/K** | 160.0(2) |
| **Crystal system** | monoclinic |
| **Space group** | *P*2_1_/*n* |
| ***a*/Å** | 8.42179(6) |
| ***b*/Å** | 22.57764(15) |
| ***c*/Å** | 8.60767(7) |
| **α/°** | 90 |
| **β/°** | 109.3386(8) |
| **γ/°** | 90 |
| **Volume/Å^3^** | 1544.35(2) |
| ***Z*** | 4 |
| **ρ_calc_g/cm^3^** | 1.348 |
| **μ/mm^‑1^** | 0.770 |
| ***F*(000)** | 664.0 |
| **Crystal size/mm^3^** | 0.23 × 0.18 × 0.11 |
| **Radiation** | CuKα (λ = 1.54184 Å) |
| **2Θ max. for data collection/°** | 160 |
| **Index ranges** | -9 ≤ *h* ≤ 10, -26 ≤ *k* ≤ 28, -10 ≤ *l* ≤ 10 |
| **Reflections collected** | 13974 |
| **Independent reflections** | 3315 [*R*_int_ = 0.0174, *R*_sigma_ = 0.0142] |
| **Data/restraints/parameters** | 3315/0/235 |
| **Goodness-of-fit on *F*^2^** | 1.040 |
| **Final *R* indexes [*I* > 2σ(*I*)]** | *R*_1_ = 0.0344, *wR*_2_ = 0.0902 |
| **Final *R* indexes [all data]** | *R*_1_ = 0.0352, *wR*_2_ = 0.0909 |
| **Largest diff. peak/hole / e Å^-3^** | 0.29/-0.25 |

**Table S3**. Fractional Atomic Coordinates (×10^4^) and Equivalent Isotropic Displacement Parameters (Å2×103) for compound **26** (named BGiii159). Ueq is defined as 1/3 of of the trace of the orthogonalised UIJ tensor.

| **Atom** | ***x*** | ***y*** | ***z*** | ***U*(eq)** |
| --- | --- | --- | --- | --- |
| O1 | 3212.0(10) | 8016.8(3) | 7895.2(9) | 27.89(19) |
| C2 | 2950.8(13) | 8390.2(5) | 6588.9(14) | 25.4(2) |
| C3 | 1416.1(13) | 8285.1(5) | 5107.0(13) | 23.9(2) |
| C4 | 1790.0(12) | 7788.2(4) | 4071.5(12) | 20.8(2) |
| N5 | 2680.0(10) | 7299.5(4) | 5130.4(10) | 19.44(19) |
| C5A | 1987.1(12) | 7129.1(4) | 6363.3(12) | 20.9(2) |
| C6 | 1093.2(14) | 6605.9(5) | 6332.6(14) | 26.6(2) |
| C7 | 593.7(15) | 6443.6(6) | 7662.0(16) | 35.0(3) |
| C8 | 972.5(17) | 6803.3(6) | 9039.4(16) | 38.9(3) |
| C9 | 1802.7(16) | 7334.4(6) | 9073.2(14) | 34.4(3) |
| C9A | 2279.7(13) | 7496.4(5) | 7739.6(13) | 24.8(2) |
| O10 | 3963.7(11) | 8778.3(4) | 6719.4(11) | 34.6(2) |
| C11 | 3121.6(13) | 6821.6(4) | 4187.8(12) | 20.7(2) |
| C12 | 4402.0(13) | 6410.9(5) | 5353.0(12) | 22.6(2) |
| C13 | 5337.2(12) | 6026.7(4) | 4516.0(12) | 19.7(2) |
| O14 | 5304.2(10) | 6086.9(3) | 3111.3(9) | 26.68(18) |
| N15 | 6340.1(11) | 5585.9(4) | 5542.3(10) | 20.49(19) |
| N16 | 6339.6(11) | 5533.3(4) | 7144.7(10) | 23.9(2) |
| C17 | 7343.5(13) | 5081.9(5) | 7761.1(13) | 24.6(2) |
| C18 | 8016.0(13) | 4840.6(4) | 6591.3(14) | 24.7(2) |
| C19 | 7360.8(12) | 5163.1(4) | 5184.0(13) | 22.1(2) |
| C20 | 7599.5(18) | 4871.9(6) | 9470.2(14) | 36.6(3) |
| C21 | 7596.4(15) | 5096.6(5) | 3554.7(15) | 30.1(3) |

**Table S4.** Anisotropic Displacement Parameters (Å2×103) for compound **26** (named BGiii159). The Anisotropic displacement factor exponent takes the form: -2π2[h2a*2U11+2hka*b*U12+…].

| **Atom** | ***U*_11_** | ***U*_22_** | ***U*_33_** | ***U*_23_** | ***U*_13_** | ***U*_12_** |
| --- | --- | --- | --- | --- | --- | --- |
| O1 | 27.5(4) | 27.9(4) | 24.4(4) | -5.7(3) | 3.4(3) | 4.3(3) |
| C2 | 24.5(5) | 20.8(5) | 30.5(5) | -7.2(4) | 8.5(4) | 4.3(4) |
| C3 | 22.2(5) | 18.9(5) | 28.5(5) | 1.8(4) | 5.5(4) | 3.8(4) |
| C4 | 21.3(5) | 18.0(5) | 20.9(5) | 2.6(4) | 3.8(4) | 0.5(4) |
| N5 | 22.4(4) | 16.6(4) | 19.2(4) | 1.4(3) | 6.8(3) | 2.6(3) |
| C5A | 19.6(4) | 22.0(5) | 21.0(5) | 4.1(4) | 6.4(4) | 6.1(4) |
| C6 | 25.0(5) | 24.4(5) | 30.6(6) | 6.4(4) | 9.7(4) | 3.7(4) |
| C7 | 30.8(6) | 34.5(6) | 44.2(7) | 16.4(5) | 18.7(5) | 6.8(5) |
| C8 | 37.9(6) | 50.8(8) | 36.4(6) | 18.4(6) | 23.6(5) | 16.9(6) |
| C9 | 36.0(6) | 44.9(7) | 25.0(5) | 3.6(5) | 13.7(5) | 16.6(5) |
| C9A | 22.7(5) | 27.0(5) | 23.9(5) | 1.5(4) | 6.5(4) | 8.1(4) |
| O10 | 30.4(4) | 27.1(4) | 45.2(5) | -12.1(4) | 11.0(4) | -4.4(3) |
| C11 | 23.9(5) | 18.8(4) | 18.7(5) | -0.9(4) | 6.1(4) | 1.4(4) |
| C12 | 27.3(5) | 21.0(5) | 18.9(5) | -0.3(4) | 6.8(4) | 4.8(4) |
| C13 | 20.4(5) | 17.2(4) | 21.2(5) | -0.3(3) | 6.6(4) | -1.5(3) |
| O14 | 34.3(4) | 26.4(4) | 22.4(4) | 3.6(3) | 13.6(3) | 4.3(3) |
| N15 | 22.5(4) | 20.2(4) | 19.9(4) | 0.6(3) | 8.5(3) | 1.8(3) |
| N16 | 28.5(5) | 24.6(4) | 18.1(4) | 1.2(3) | 7.0(3) | 2.9(3) |
| C17 | 25.1(5) | 22.0(5) | 23.4(5) | 1.1(4) | 3.8(4) | -0.4(4) |
| C18 | 21.3(5) | 19.3(5) | 32.9(6) | 2.3(4) | 8.3(4) | 1.4(4) |
| C19 | 20.2(5) | 18.1(5) | 30.3(5) | -0.2(4) | 11.7(4) | -0.7(4) |
| C20 | 47.6(7) | 33.4(6) | 23.9(6) | 6.5(5) | 5.4(5) | 5.7(5) |
| C21 | 36.5(6) | 25.6(5) | 36.9(6) | 2.6(4) | 23.9(5) | 4.8(4) |

**Table S5.** Bond Lengths for compound **26** (Named BGiii159).

| **Atom** | **Atom** | **Length/Å** |  | **Atom** | **Atom** | **Length/Å** |
| --- | --- | --- | --- | --- | --- | --- |
| O1 | C2 | 1.3637(14) |  | C9 | C9A | 1.3850(16) |
| O1 | C9A | 1.3947(14) |  | C11 | C12 | 1.5206(13) |
| C2 | C3 | 1.5040(15) |  | C12 | C13 | 1.5057(14) |
| C2 | O10 | 1.2020(14) |  | C13 | O14 | 1.2079(13) |
| C3 | C4 | 1.5289(14) |  | C13 | N15 | 1.4107(13) |
| C4 | N5 | 1.4690(12) |  | N15 | N16 | 1.3845(12) |
| N5 | C5A | 1.4229(13) |  | N15 | C19 | 1.3860(13) |
| N5 | C11 | 1.4699(12) |  | N16 | C17 | 1.3184(14) |
| C5A | C6 | 1.3964(15) |  | C17 | C18 | 1.4174(15) |
| C5A | C9A | 1.3994(15) |  | C17 | C20 | 1.4918(15) |
| C6 | C7 | 1.3927(16) |  | C18 | C19 | 1.3635(15) |
| C7 | C8 | 1.384(2) |  | C19 | C21 | 1.4876(15) |
| C8 | C9 | 1.383(2) |  |  |  |  |

**Table S6.** Bond Angles for compound **26** (Named BGiii159).

| **Atom** | **Atom** | **Atom** | **Angle/˚** |  | **Atom** | **Atom** | **Atom** | **Angle/˚** |
| --- | --- | --- | --- | --- | --- | --- | --- | --- |
| C2 | O1 | C9A | 120.64(8) |  | C9 | C9A | C5A | 121.81(11) |
| O1 | C2 | C3 | 117.42(9) |  | N5 | C11 | C12 | 109.71(8) |
| O10 | C2 | O1 | 117.25(10) |  | C13 | C12 | C11 | 113.87(8) |
| O10 | C2 | C3 | 125.33(11) |  | O14 | C13 | C12 | 124.88(9) |
| C2 | C3 | C4 | 108.97(8) |  | O14 | C13 | N15 | 121.11(9) |
| N5 | C4 | C3 | 110.53(8) |  | N15 | C13 | C12 | 113.98(8) |
| C4 | N5 | C11 | 112.29(8) |  | N16 | N15 | C13 | 119.12(8) |
| C5A | N5 | C4 | 114.51(8) |  | N16 | N15 | C19 | 111.55(8) |
| C5A | N5 | C11 | 117.03(8) |  | C19 | N15 | C13 | 129.31(9) |
| C6 | C5A | N5 | 124.49(9) |  | C17 | N16 | N15 | 104.81(8) |
| C6 | C5A | C9A | 117.39(10) |  | N16 | C17 | C18 | 111.41(9) |
| C9A | C5A | N5 | 118.07(9) |  | N16 | C17 | C20 | 120.44(10) |
| C7 | C6 | C5A | 120.98(11) |  | C18 | C17 | C20 | 128.11(10) |
| C8 | C7 | C6 | 120.17(12) |  | C19 | C18 | C17 | 106.65(9) |
| C9 | C8 | C7 | 119.87(11) |  | N15 | C19 | C21 | 124.43(9) |
| C8 | C9 | C9A | 119.65(12) |  | C18 | C19 | N15 | 105.57(9) |
| O1 | C9A | C5A | 121.07(9) |  | C18 | C19 | C21 | 129.99(10) |
| C9 | C9A | O1 | 116.84(10) |  |  |  |  |  |

**Table S7.** Torsion Angles for compound **26** (Named BGiii159)**.**

| **A** | **B** | **C** | **D** | **Angle/˚** |  | **A** | **B** | **C** | **D** | **Angle/˚** |
| --- | --- | --- | --- | --- | --- | --- | --- | --- | --- | --- |
| O1 | C2 | C3 | C4 | -81.04(11) |  | C9A | C5A | C6 | C7 | -3.29(15) |
| C2 | O1 | C9A | C5A | 44.03(13) |  | O10 | C2 | C3 | C4 | 99.29(12) |
| C2 | O1 | C9A | C9 | -141.96(10) |  | C11 | N5 | C5A | C6 | -26.79(14) |
| C2 | C3 | C4 | N5 | 43.69(11) |  | C11 | N5 | C5A | C9A | 150.27(9) |
| C3 | C4 | N5 | C5A | 45.58(11) |  | C11 | C12 | C13 | O14 | 10.96(15) |
| C3 | C4 | N5 | C11 | -177.81(8) |  | C11 | C12 | C13 | N15 | -171.12(8) |
| C4 | N5 | C5A | C6 | 107.68(11) |  | C12 | C13 | N15 | N16 | 1.95(13) |
| C4 | N5 | C5A | C9A | -75.26(11) |  | C12 | C13 | N15 | C19 | -179.64(9) |
| C4 | N5 | C11 | C12 | 165.71(8) |  | C13 | N15 | N16 | C17 | 178.45(9) |
| N5 | C5A | C6 | C7 | 173.79(10) |  | C13 | N15 | C19 | C18 | -178.69(10) |
| N5 | C5A | C9A | O1 | 0.48(14) |  | C13 | N15 | C19 | C21 | 0.19(16) |
| N5 | C5A | C9A | C9 | -173.23(9) |  | O14 | C13 | N15 | N16 | 179.96(9) |
| N5 | C11 | C12 | C13 | -161.94(8) |  | O14 | C13 | N15 | C19 | -1.63(16) |
| C5A | N5 | C11 | C12 | -58.86(11) |  | N15 | N16 | C17 | C18 | 0.55(12) |
| C5A | C6 | C7 | C8 | 0.33(17) |  | N15 | N16 | C17 | C20 | -177.26(10) |
| C6 | C5A | C9A | O1 | 177.76(9) |  | N16 | N15 | C19 | C18 | -0.18(11) |
| C6 | C5A | C9A | C9 | 4.05(15) |  | N16 | N15 | C19 | C21 | 178.69(9) |
| C6 | C7 | C8 | C9 | 2.04(18) |  | N16 | C17 | C18 | C19 | -0.68(12) |
| C7 | C8 | C9 | C9A | -1.33(18) |  | C17 | C18 | C19 | N15 | 0.49(11) |
| C8 | C9 | C9A | O1 | -175.75(10) |  | C17 | C18 | C19 | C21 | -178.30(11) |
| C8 | C9 | C9A | C5A | -1.79(17) |  | C19 | N15 | N16 | C17 | -0.23(11) |
| C9A | O1 | C2 | C3 | 11.89(13) |  | C20 | C17 | C18 | C19 | 176.91(11) |
| C9A | O1 | C2 | O10 | -168.42(9) |  |  |  |  |  |  |

**Table S8.** Hydrogen Atom Coordinates (Å×104) and Isotropic Displacement Parameters (Å2×10^3^) for compound **26** (Named BGiii159).

| **Atom** | ***x*** | ***y*** | ***z*** | ***U*(iso)** |
| --- | --- | --- | --- | --- |
| H3A | 1194(17) | 8642(6) | 4479(17) | 28(3) |
| H3B | 473(18) | 8182(6) | 5485(16) | 28(3) |
| H4A | 722.14 | 7640.13 | 3278.24 | 25 |
| H4B | 2489.11 | 7944.29 | 3438.57 | 25 |
| H6 | 821.77 | 6356.96 | 5390.88 | 32 |
| H7 | -8.54 | 6085.04 | 7623.53 | 42 |
| H8 | 662.92 | 6685.74 | 9959.53 | 47 |
| H9 | 2044.23 | 7586.67 | 10006.16 | 41 |
| H11A | 3613(16) | 7018(6) | 3425(16) | 24(3) |
| H11B | 2140(17) | 6602(6) | 3498(16) | 23(3) |
| H12A | 5253(19) | 6661(7) | 6168(18) | 35(4) |
| H12B | 3893(18) | 6160(6) | 5981(18) | 31(3) |
| H18 | 8775.41 | 4517.14 | 6755.82 | 30 |
| H20A | 7281.78 | 5186.78 | 10094.05 | 55 |
| H20B | 8785.97 | 4769.12 | 10008.61 | 55 |
| H20C | 6898.88 | 4521.95 | 9430.78 | 55 |
| H21A | 6511.37 | 5007.79 | 2714.74 | 45 |
| H21B | 8386.01 | 4772.5 | 3605.84 | 45 |
| H21C | 8046.88 | 5465.87 | 3270.29 | 45 |
