# Supplementary material for: Synthesis of novel N-substituted β-amino acid derivatives bearing 2-hydroxyphenyl moieties as promising antimicrobial candidates targeting multidrug-resistant Gram-positive pathogens
Source: PLoS One. 2025 Jun 12;20(6):e0311715. doi: 10.1371/journal.pone.0311715 (PMC12161524; doi:10.1371/journal.pone.0311715)
Supplement: Attachment — Submitted filename: Review20240930.PONE-D-24-42309.docx [file pone.0311715.s002.docx]

Review “ Synthesis of Novel N-Substituted β-Amino Acid Derivatives Bearing 2-Hydroxyphenyl Moieties as Promising Antimicrobial Candidates Targeting Multidrug-Resistant Gram-Positive Pathogens“

Lines 51-53 Check citations. *Enterococcus* species or *Enterococcus faecium*? It is important.

Line 57 Check *Enterococcus* or *Enterococcci* or *Enterococcus faecium*. What do you mean „Enterococcus“?

Line 441 Check the name *E. clocea* or *E. cloacae* ? Write the full name of this pathogen when you mention it for the first time.

Line 442 Institute of Infectious Diseases and Pathogenic Microbiology collection. Where is it?

Line 446 What about fungal strains? It must be described.

Line 453 A producer of 96-well microplates is not mentioned.

Line 455 A producer of CAMHB is not mentioned.

Line 456 A producer of RMPI/MOPS media is not mentioned.

Line 463 „tested drug“ or tested compound?

Line 466 The control of antibacterial and antifungal agents is not described. The producers of antibiotics must be mentioned.

Line 521 You must write *Enterococcus faecalis*. Do not us Italic for spp. !

Line 522 spp. but not spp

Line 522 *E. cloacae* but not *Enterobacter* spp

Line 530 You used bacterial strains and isolates, but not only isolates. The title of the table 1 must be improved.

Table 1 Explain, what „128>“ means. You write „128>“ when the compound concentration of 128 mg/mL showed no antimicrobial activity and the higher concentration equal to 256 mg/mL was not tested. It would be better to write that the results were negative. „128 >“ is an interpretation of your results? In my opinion, the presentation of data in the table 1 is subject to discussion or correction. Furthermore, in the abstract, you write that „one of the compounds demonstrated activity against Gram-negative pathogens“.

Explain why MBCs of compounds were not determined.

Line 614 „Enterococcus species.,“? Check grammar! Isn‘t the member of ESKAPE group only *Enterococcus faecium*? Is *E. faecalis* also a member of ESKAPE group?

Line 678 *Staphylococcus* but not Staphylococcus

Line 684 „Collectively, in this study,“ The beginning of conclusions does not seem good
