# Supplementary material for: Synthesis of novel N-substituted β-amino acid derivatives bearing 2-hydroxyphenyl moieties as promising antimicrobial candidates targeting multidrug-resistant Gram-positive pathogens
Source: PLoS One. 2025 Jun 12;20(6):e0311715. doi: 10.1371/journal.pone.0311715 (PMC12161524; doi:10.1371/journal.pone.0311715)
Supplement: Attachment — Submitted filename: Response to the reviewer.docx [file pone.0311715.s004.docx]

**To the Editor of PLOS One,

To the reviewer,


Dear reviewer,**


On behalf of all authors, I would like to extend our gratuity for your valueble comments and suggestions aimed to improve this paper. We have incorporated all changes to the revised manuscript. Please find bellow our detailed response to the comments.

We hope that the revised manuscript will be suitable for the readership of PLOS One.

We thank you,

**Sincerely,**

**Povilas Kavaliauskas**

***Response to the reviewer‘s comments:***

Review “ Synthesis of Novel N-Substituted β-Amino Acid Derivatives Bearing 2-Hydroxyphenyl Moieties as Promising Antimicrobial Candidates Targeting Multidrug-Resistant Gram-Positive Pathogens“

**Lines 51-53 Check citations. *Enterococcus* species or *Enterococcus faecium*? It is important.**

**Line 57 Check *Enterococcus* or *Enterococcci* or *Enterococcus faecium*. What do you mean „Enterococcus“?**

*We thank the reviewer for great comments and we apologize for the comfussion. We have clarified our statemts.*

**Line 441 Check the name *E. clocea* or *E. cloacae* ? Write the full name of this pathogen when you mention it for the first time.**

*We have incorporated the changes into manuscript, materials and methods section. Together with Enterobacter isolate, we also provided full names of used pathogens.*

**Line 442 Institute of Infectious Diseases and Pathogenic Microbiology collection. Where is it?**

*Our apologies for missing this information. Institute of Infectious Diseases and Pathogenic Microbiology is a colaborator from Prienai, Lithuania. We have provided this information to the mansucript.*

**Line 446 What about fungal strains? It must be described.**

*We apologize for the editing mistake and not including these descriptions. We have now provided this information to the manuscript. In addition to that, we noticed that the strain numbers were also missing, in table S1, therefore we have updated Table S1.

Bellow is the requested information (L448-452:

Candida albicans AR-0761 and Candida auris AR-0381 were obtained from ARisolate bank at CDC Atlanta. Aspergillus fumigatus CEA10, Cuninghamella bertholiatiae NIH-182, and Rhizopus delamar 1221 were kindly provided by Dr. Vidmantas Petraitis (Weill Cornell Medicine of Cornell University). Candida krusei ATCC 32196 were obtained from ATCC.*

**Line 453 A producer of 96-well microplates is not mentioned.**

**Line 455 A producer of CAMHB is not mentioned.**

**Line 456 A producer of RMPI/MOPS media is not mentioned.**

*We thank for the great comment. We have provided the requested information.
Deep 96-well plates were purchased from* Nunc, Thermo Scientiffic, Waltham, United States*.

CABH was obtained from ThermoScientiffic while RPMI/MOPS was obtained from* Criterion, Hardy Diagnostics, West McCoy Lane Santa Maria.

**Line 463 „tested drug“ or tested compound?**

*We have addressed the comment and clarified that this was indeed a tested compound.*

**Line 466 The control of antibacterial and antifungal agents is not described. The producers of antibiotics must be mentioned.**

*We apologize for missing this information. We have provided the sources of these materials.

Vancomycin hydrochloride and gentamycin sulphate were dissolved in sterile deionized water, while meropenem and cefazolin were dissolved in DMSO (MedChemExpress, Deer Park, United Stated).*

*Amphotericin B, fluconazole (Sigma, St. Louis, United States), posaconazole, and voriconazole (MedChemExpress, Deer Park, United Stated) were dissolved in DMSO and further serially diluted using RPMI/MOPS broth.*

**Line 521 You must write *Enterococcus faecalis*. Do not us Italic for spp. !**

**Line 522 spp. but not spp**

**Line 522 *E. cloacae* but not *Enterobacter* spp**

**Line 530 You used bacterial strains and isolates, but not only isolates. The title of the table 1 must be improved.**

*We have addressed these issues and incorporated the changes in the manuscript.*

**Table 1 Explain, what „128>“ means. You write „128>“ when the compound concentration of 128 mg/mL showed no antimicrobial activity and the higher concentration equal to 256 mg/mL was not tested. It would be better to write that the results were negative. „128 >“ is an interpretation of your results? In my opinion, the presentation of data in the table 1 is subject to discussion or correction. Furthermore, in the abstract, you write that „one of the compounds demonstrated activity against Gram-negative pathogens“.**

*We thank the reviewer for great comment. We first of all revised the table 1, as suggested and changed the previous value (128>) to the actual value of >128.
This shows that the tested compounds did not showed activity at the highest tested concentration (128 microg/mL). This value is provided based on CLSI guidelines, therefore we would kindly ask the reviewer to maintain the current corrected way.*

**Explain why MBCs of compounds were not determined.**

*We thank you for the comment. We agree with the reviewer that MBC would highly benefit the readers of the paper. As commented by the reviewer, we performed the additional experiments and determined MBC for all synthesized compounds using E. faecalis* AR-0781 and *S. aureus* TCH-1516 strains (Table S2).
 **Table S2.** The minimal bactericidal concentration (MBC) of *N*-substituted *β*-amino acid derivatives **2-26** against drug-resistant Gram-positive pathogens.

|  | **Minimal bactericidal concentration (µg/ml)** | |
| --- | --- | --- |
| **Compound** | ***E. faecalis* AR-0781^A^** | ***S. aureus* TCH-1516^B^** |
| **2** | >128 | >128 |
| **3** | >128 | >128 |
| **4** | >128 | >128 |
| **5** | >128 | >128 |
| **6** | >128 | >128 |
| **7** | >128 | >128 |
| **8** | >128 | 64 |
| **9** | 16 | 16 |
| **10** | >128 | >128 |
| **11** | >128 | >128 |
| **12** | >128 | 32 |
| **13** | >128 | >128 |
| **14** | >128 | 64 |
| **15** | >128 | >128 |
| **16** | >128 | 128 |
| **17** | >128 | 8 |
| **18** | >128 | 32 |
| **19** | >128 | >128 |
| **20** | >128 | >128 |
| **21** | >128 | >128 |
| **22** | >128 | >128 |
| **23** | >128 | >128 |
| **24** | >128 | 32 |
| **25** | 64 | 64 |
| **26** | 16 | 8 |
| **Vancomycin** | >128 | 4 |
| **Gentamycin** | 64 | 128 |
| **Meropenem** | 8 | 16 |
| **Cefazolin** | 32 | >128 |

A. Vancomycin-resistant *E. faecalis* harboring *tet(L), tet(M), VanA* resistance genes.

B. Meticillin-Resistant *S. aureus* USA300 lineage harboring *mecA* and Panton-Valentine leucocidin *pvl.*

**Line 614 „Enterococcus species.,“? Check grammar! Isn‘t the member of ESKAPE group only *Enterococcus faecium*? Is *E. faecalis* also a member of ESKAPE group?**

*Our apologies for grammar mistakes. We have clarified the taxonomy and ESKAPE clarification. We have not used VRE E. faecium for the studydue to lack of the isolates in our disposition. Despite that, we have access to VRE E. faecalis. Based on the literature, E. faecalis also becoming rapidly resistant to vancomycin and other antimicrobials, therefore we used it for the study.

We have incorporated changes.*

**Line 678 *Staphylococcus* but not Staphylococcus**

*Our apologies for the mistake. We have addressed it and changes are incorporated to the mansucript.*

**Line 684 „Collectively, in this study,“ The beginning of conclusions does not seem good**

*We have revised the conclussion section to avoid the mentioned statement.*

*In this study, we report the synthetic pathways for the generation of novel libraries of 3,3'-((2-hydroxyphenyl)azanediyl)dipropionic acid derivatives, incorporating functional groups such as ester, hydrazine, hydrazones, benzimidazole, dimethylpyrrole, dimethylpyrazole, and oxadezipine moieties. These compounds demonstrated promising structure-depended antimicrobial activity against multidrug-resistant Gram-positive pathogens, notably methicillin-resistant Staphylococcus aureus (MRSA) and vancomycin-resistant Enterococcus (VRE). Structure-activity relationship (SAR) analysis revealed that electron-withdrawing nitro-aromatic and heterocyclic substituents significantly enhanced antimicrobial efficacy. Compound 26 exhibited broad-spectrum antibacterial and antifungal activity, demonstrating the potential of this scaffold for further hit-to-lead optimization. While these initial results are encouraging, further studies are needed to understand the activity of compound 26 and various compound 26-based derivatives against a broader range of drug-resistant Gram-positive isolates. These findings highlights that N-substituted β-amino acid derivatives could be explored as a promising platform for the development of novel antimicrobial agents targeting predominantly multidrug-resistant gram-positive pathogens.*
