# Supplementary material for: Synthesis of novel N-substituted β-amino acid derivatives bearing 2-hydroxyphenyl moieties as promising antimicrobial candidates targeting multidrug-resistant Gram-positive pathogens
Source: PLoS One. 2025 Jun 12;20(6):e0311715. doi: 10.1371/journal.pone.0311715 (PMC12161524; doi:10.1371/journal.pone.0311715)
Supplement: Attachment — Submitted filename: Reviewers comments_rev2_2025-02-08.docx [file pone.0311715.s005.docx]

**To the Editor of PLOS One,**

**To the reviewers**

Dear Sir/Madam,


On behalf of all authors I would like to thank the reviewers for their expertise and valuable comments aimed to improve this manuscript.
We have responded to all comments and incorporated the changes to the manuscript.

Bellow please find the detailed response to the comments.

We hope that the current, updated form of the manuscript will meet the merit of PLOS One journal.

Sincerely,

Povilas Kavaliauskas

**Reviewer #2**:

This manuscript is devoted to the development of new antimicrobial drugs. The relevance of this issue is undeniable and constant, since it is necessary to continuously update the pool of these drugs due to the resistance to active substances developed by pathogenic organisms. The authors focused their attention on N-substituted β-amino acid derivatives bearing a 2-hydroxyphenyl moiety, synthesizing a number of compounds containing this fragment and belonging to several very different classes of organic compounds, including heterocyclic ones. Having determined their activity in relation to several types of pathogenic microorganisms, they found that the substituted 1,3,4-oxadiazole-2(3H)-thione showed the greatest activity.

In this regard, I have a wish for the authors: it is worth discussing in more detail which fragment determines the activity in this case - β-amino acid or 1,3,4-oxadiazole-2(3H)-thione.

From a chemical point of view, the work is of particular interest in that the authors did not limit themselves to one or two reactions, but demonstrated the richness of organic synthesis and the diversity of chemical properties of carboxylic acids. Personally, I found it interesting to read the manuscript. The experiment was performed well. I have no doubts about the structure of the obtained compounds, since they are almost completely characterized, and the quality of the presented NMR spectra is quite high.

Several comments on the description of the results are given below:
**1. In Scheme 3 and in the text that describes it, the initial dicarbonyl compounds (acetylacetone and acetonylacetone) are confused.**

*We are thankful for the comment. We have corrected it.*

**2. It would be very good if the authors could explain why in the 1H NMR spectra of dihydrazides 7-14 there are 3 signals of the phenol proton and 3-4 signals of the NH protons, while in the 13C NMR spectra of some of these compounds there are 2 signals of carbonyl carbon atoms.**

*We are thankful for the comment. This is related the restricted rotation around the CONH led to the formation in an isomeric mixture of hydrazones where Z isomer predominates. The obtained hydrazones 7−14 show double sets of resonances for the N=CH and CONH fragment protons with the intensity ratio of 0.3:0.7 (^1^H NMR). No formation of geometrical isomers was observed.*

**There are two questions here: a) why are there several signals at all, despite the apparent symmetry of these compounds;**

*We are thankful for the comment. As we mentioned before.* *This is related the restricted rotation around the CONH led to the formation in an isomeric mixture of hydrazones where Z isomer predominates. The obtained hydrazones 7−14 show double sets of resonances for the N=CH and CONH fragment protons with the intensity ratio of 0.3:0.7 (1H NMR). No formation of geometrical isomers was observed.*

**b) why the number of forms according to 1H and 13C NMR data is sometimes different? It is desirable to add a discussion of this fact to the discussion section devoted to these compounds.**

*We are thankful for the comment. We have added this point in the discussion section.*
**3. In the description of the fluorine-substituted derivative (compound 8), the 19F NMR spectrum should be added, and either the signals of carbon atoms with C-F coupling constants should be indicated, or the spectrum with fluorine decoupling should be registered. Currently, the 13C NMR spectrum does not formally correspond to the proposed structure, since it contains 29 signals of aromatic protons, which exceeds all reasonable limits.**

*We are thankful for the comment. We have performed the additional experiments, where we recorded 19F NMR. THe results are added 19F NMR in the compound* ***8*** *experimental part description.*

**4. The description of the synthetic methods contains many typos and language errors. For example, in the description of the synthesis of compound 3, for some reason, the present tense is used instead of the past tense, as in the description of compound 2 (and as it should be). Moreover, this is far from the only example of a tense discrepancy, sometimes even within a single sentence. Examples of other inaccuracies (not exhaustive):**

*We are thankful for the comment. We have corrected it.*
**- Line 138. "the reaction mixture 20 minutes stirred" should be changed to "the reaction mixture was stirred for 20 minutes".**

*We are thankful for the comment. We have corrected it.*
**- Line 154. "the residuse neutralized" should be changed to "the residue was neutralized".
The authors should proofread this section more carefully.**

*We are thankful for the comment. We have corrected it.*

**Reviewer #3**:

- **The study has been well designed, organized, worked and prepared. The topic is important and actual. It seems the continuation of the previous work (ref.28) in order to investigate and compare the effects of the (various) substituent groups in different positions on the drug-resistant patogens.**

**- In the results part, H-NMR values are in 2 decimals, but also C-NMR values are in 2 decimals. However, the C-NMR values should be written in 1 decimal in a scientific paper.**

*We are thankful for the comment. We have corrected it.*

**- In some H-NMR and C-NMR results, the numbers of protons and carbons are inconsistent with the spectra/structures, this should be checked.**

*We are thankful for the comment. We have have* checked and corrected it.

**- There are some additional/extra peaks for the H-NMR and C-NMR results, are they impurities? All purifications in the work were performed by crystallization/crystals and any other methods (e.g. column chromatography) were apply to purify the compounds, was this enough for a good purification? Because, especially for the biological activity tests purity/impurities are of high importance.**

*We agree with the reviewers comment regarding potential impurities or break down products. Based on your available purification methods, we aimed to obtain compounds as pure as possible. With that being said, we take this as a potential limitation of this study. With methods available to our group, we obtained compounds with reasonable purity of compounds, as confirmed by NMR, elemental analysis, and mass spectrometry data.*

- Mass spectroscopy analyses are present for some compounds, not for all, is there a specific reason for that?

*Due to the institutional changes as well as maintenance problems, the mass spectrometer is no longer available to us and our collaborators. Therefore, we are unable to record this data. We hope that NMR spectrums and other supporting constants will be sufficient to prove the identityof the synthesized compounds.*

**- Are the synthesized compounds new? If yes, this was not really emphasized in the manuscript and abstract, this is important and should be mentioned in the appropriate parts of the manuscript. If no, then the related references must be given.**

*We are thankful for the comment. Yes, all synthesized compounds are new, it were mentioned in the corresponding parts of the manuscript.*

- The preparation purposes of nearly all compound were well explained in the synthesis part except compound-6. It should also be mentioned in this part.

*We are thankful for the comment. We have corrected it.*

**- Between the lines 582 and 603, the same issue was discussed and repeated twice, this should be checked and rearranged.
- Line 693: resistant; twice.**

*We have addressed this comment and incorporated the changes.*

**Reviewer #4**:

The article entitled “Synthesis of Novel N-Substituted β-Amino Acid Derivatives Bearing 2-Hydroxyphenyl Moieties as Promising Antimicrobial Candidates Targeting Multidrug-Resistant Gram-Positive Pathogens” describes the synthesis and biological evaluation of N-Substituted β-Amino Acid Derivatives with a 2-hydroxyphenyl moeity as the antimicrobial agents. The manuscript would be of general interest to the researchers of this field. Before recommending for publication, the authors should consider and incorporate in the present form of the manuscript of some concerns and comments that need to be addressed.

Some comments and corrections for authors:

1. **Overally, the manuscript has some punctuation and grammatical errors and needs to be corrected (i.e., there must be comma before and in all mns when mention about over two parameters). Please run throughout the mns.**

*We are thankful for the comment. We have corrected it.*

**2. A figure showing the importance of N-substitution in β-amino acids in med chem from literature should be added to the intro section.**

*We are thankful for the comment. We have corrected it.*

**Figure 1.** Pharmaceuticals containing *β*-amino acid structures.

**3. The reaction pathway is clear. But some of compounds were not fully characterized. Some molecules do not have FTIR analysis, others do not have HRMS analysis**.

*We are thankful for the comment. But we disagree with the first statement, we have the FTIR analysis for all synthesized compounds. Due to institutional challenges and the broken instrument, we are not able to obtain HRMS data.*
 **4. The authors must define the unknow/known compounds in the mns.**

*We are thankful for the comment. We have corrected it.*

**5. I’d prefer to see the X-ray single analyses of unexpected products such as 3, 6, and 24.**

*We are thankful for the comment. To address this, we have performed X ray cristalography of compound 24. We are providing this data as Figure 1.*

**Figure 1.** The ORTEP diagram of analysed compound 24 showing the numbering scheme used in this study.

The analytical data is provided as a supplement and can be found as:

**Table S2.** Crystal data and structure refinement for compound **24** (named BGiii159).

**Table S3**. Fractional Atomic Coordinates (×10^4^) and Equivalent Isotropic Displacement Parameters (Å2×103) for compound **24** (named BGiii159). Ueq is defined as 1/3 of of the trace of the orthogonalised UIJ tensor.

**Table S4.** Anisotropic Displacement Parameters (Å2×103) for compound **24** (named BGiii159). The Anisotropic displacement factor exponent takes the form: -2π2[h2a*2U11+2hka*b*U12+…].

**Table S5.** Bond Lengths for compound **24** (Named BGiii159).

**Table S6.** Bond Angles for compound **24** (Named BGiii159).

**Table S7.** Torsion Angles for compound **24** (Named BGiii159)**.**

**Table S8.** Hydrogen Atom Coordinates (Å×104) and Isotropic Displacement Parameters (Å2×10^3^) for compound **24** (Named BGiii159).

**6. “ml” must be corrected as “mL”. Please check and correct throughout the mns.**

*We have incorporated the changes.*

**7. The data of carbon NMR values should be one decimal after point.**

*We have incorporated the changes.*

8**. I strongly recommend to the authors to support their findings with in silico methods and further studies such as time-kill assay and/or biofilm formation especially for 9 and 26.**

*We are thankful for the reviewer for their great suggestion. As requested, we performed time-kill assay t propose the mechanisms of action. We are introducing this data to figure 2.*

**Figure 2. The time-kill kinetics of compounds 9 and 26 against multidrug-resistant *E. faecalis* AR-0781 (panel A) and *S. aureus* TCH-1516 (panel B) isolates.** The bacterial strains were exposed to sub-MIC, MIC, and 1× MIC concentrations of each compound and incubated for 24 hours. At designated time points, the microbial cultures were aliquoted, serially diluted, and plated on sheep blood agar plates. The colonies were counted, and bacterial burden (expressed as log₁₀ CFU/mL) was calculated. The data are presented as the mean ± SD of three experimental replicates.
